# Human perception and response to sound from unmanned aircraft systems within ambient acoustic environments

**DOI:** 10.1038/s44384-024-00001-6

**Published:** 2025-02-12

**Authors:** Michael J. B. Lotinga, Marc C. Green, Antonio J. Torija

**Affiliations:** Acoustics Research Centre, University of Salford, Salford, Greater Manchester UK

**Keywords:** Health care, Engineering, Psychology

## Abstract

Potential opportunities for unmanned aircraft systems (UAS) to offer societal benefits are accompanied by noise impact risks. Accordingly, it is important to develop greater understanding of perception and response to UAS sound. A laboratory listening experiment was undertaken to address this aim by investigating psychoacoustics of UAS sound exposure. The experiment incorporated contextual auditory and soundscape factors by embedding spatially-rendered UAS sounds within urban acoustic environments. The UAS covered varying aircraft designs, operating modes and numbers of flights. The experiment was focussed on determining noticeability and noise annoyance. The results indicate that annoyance responses were influenced by UAS type, operational mode, sound characteristics, quantities of flights, and the ambient acoustic environments in which UAS events occurred. Annoyance also appeared to have associations with personal attitude towards advanced air mobility technology, and with classification of residence area. Noticeability appeared to be influenced by UAS type, operating mode, loudness and ambient environment.

## Introduction

Unmanned aircraft systems (UAS, also known as unmanned aerial vehicles or ‘drones’) are expected to be deployed in considerable numbers in the near future, to fulfil a wide range of mobility and logistics roles^1^. Alongside these opportunities, and the potential economic benefits, arise risks of adverse impact from noise introduced by this emerging technology^2^. Conventional civil aviation noise remains a major concern in terms of public health and wellbeing^3^; the emergence of a new generation of aircraft will require careful management to ensure that the incorporation of UAS and other advanced air mobility (AAM) vehicles into the airspace avoids exacerbating these issues. The REFMAP (Reducing Environmental Footprint through Multi-scale Aviation Planning) project objective is to develop an online platform enabling aviation planners and operators to optimise flightpath routing for both conventional aircraft and UAS. Among other constraints, including airflow and greenhouse gas emissions, the tool will support optimisation for noise impact.

A psychoacoustic study is being undertaken to support the development of the REFMAP platform with predictive models, and to further understanding of perception and response to sound from UAS. Recent reviews have summarised relevant research that informs expectations in relation to developing suitable research questions: Schäffer et al.^4^ noted studies identifying tonality, roughness, fluctuation strength and sharpness as potential contributors to subjective responses, alongside loudness. They also noted the spectral features of small multicopter UAS as comprising clearly distinguishable low- and mid-frequency tones, along with considerable high-frequency broadband energy, which would be consistent with the apparent importance of tonality and sharpness sound qualities for perception. Considering subsequent studies, Lotinga et al.^5^ supported these observations, while highlighting research suggesting impulsiveness could also play a role in perception, for rotor designs in which blade-wake interaction becomes important^6^. Lotinga et al.^5^ also considered research investigating combinations of UAS sound with ambient acoustic environments and visual contexts, noting study results indicating greater adverse impact from UAS sound within calmer, quieter soundscapes than in busier, noisier ones. Individual differences, in terms of personal or contextual factors, have also been identified as potentially influential in responses to UAS sound^5^. These include attitudes towards AAM technology^7^, contextual cues (i.e., response framing) and geographic location^8^. Research gaps highlighted in these reviews include:

- Systematic studies of the effects of varying designs, weights, operations, speeds and payloads on responses.
- Developing understanding of the role played by personal and contextual factors (such as ambient soundscapes and individual differences) influencing responses.
- Investigation of more complex operating scenarios beyond single flight events.
- Improvement of models and metrics by establishing sound qualities of importance in relation to UAS perception.

More-recent experimental research has shown that judgements of different UAS flight operational modes vary, with flyby (or flyover) operation tending to be less annoying than other modes, such as takeoff and landing^9^. Further analysis by Green et al.^9^ also indicated impulsiveness as a potentially important sound quality in determining perception and response to UAS, which was observed to be elevated during landing operations; this was postulated to be associated with blade-vortex interaction.

Recent developments in AAM research have also included investigating subjective responses to multiple movements of concept rotorcraft within an experimental context^10^: analysis showed that either averaged sound energy or numbers of vehicle events were more or less influential, depending on the event quantities—as events increased in quantity (at equal overall energy), a ‘step-change’ in responses was observed that indicated a change in perception from ‘frequent sounds’ to ‘constant’ sounds; this change was observed to occur at approximately 2 vehicle events per minute, for the auralised AAM quadrotor aircraft.

To investigate and make advances on these aspects, a controlled listening experiment has been undertaken in laboratory conditions, aiming to address the following research questions:

1. How are perception and affective responses to UAS sounds influenced by the UAS sonic characteristics?
2. How do varying UAS design types (size/weight, rotor configuration) and operational modes affect noticeability and responses?
3. How do different ambient soundscapes influence noticeability and responses to UAS sounds?
4. What connections are there between noticeability and responses to UAS sounds?
5. How do responses to multiple UAS sounds vary with changing event quantities, while overall UAS sound energy is held constant?
6. How do personal factors influence individual responses?

The experiment (as detailed in the Methods section) was carried out in two parts: Part A was focussed on the aspects of the research questions addressing perception and responses to different UAS types, flight operations and ambient acoustic environments. Part B was focussed on judgements of multiple UAS events. Participants provided perceptual and affective judgements of several spatially-rendered audio scenes covering a varied range of UAS sounds embedded within ambient acoustic environments.

The results presented are focussed on two aspects of perception and response: (i) noticeability of UAS sounds within ambient environments, which we define here as the proportion of participants assigned a ‘UAS noticed’ classification from a source typology (see Participants section), and (ii) noise annoyance.

Given the aforementioned research questions and previous findings, our hypotheses included the following:

- UAS loudness or sound level is positively associated with noticeability and annoyance (research question 1).
- UAS sound characteristics other than loudness, such as tonal quality, spectral skew, impulsivity or temporal modulation, also have some influence on noticeability and annoyance (research questions 1 & 2).
- UAS sounds are more annoying and noticeable in a quieter, calmer acoustic environment than in a busier, noisier one (research question 3).
- More noticeable UAS sounds are associated with greater annoyance (research question 4).
- Landing or takeoff operational modes are associated with greater annoyance than flybys (research questions 1 & 2).
- Different UAS design types (size and rotor configurations) have varying associations with noticeability and/or annoyance (research questions 1 & 2).
- Greater UAS sound event quantities (at equal sound energy) are associated with increased annoyance up to a point, with further increases in event quantities producing negligible changes in response (research question 5).

## Results

This section presents results from exploratory and confirmatory data analyses (these terms are used here in the same sense as proposed by Fife and Rodgers^11^—see Supplementary Note 1 in the Supplementary information for further details) of the annoyance and noticeability judgements of the experiment stimuli provided by the participant sample. See the Methods section for details of the experiment methodology and execution.

### Part A

**Fig. 1:**
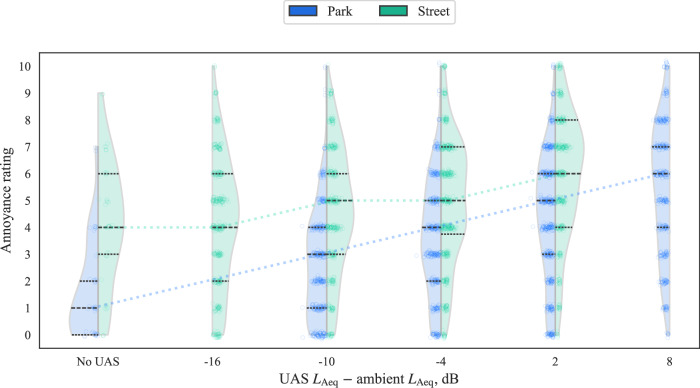
Part A results (*n* = 41): Annoyance ratings by UAS Δ*L*_Aeq_ relative to ambient; (violin plots show kernel density and black quartile lines, coloured dotted lines track median values, data points *xy*-jittered).

The annoyance response results of Part A are presented in Fig. 1 grouped by the difference in time-averaged sound levels (Δ*L*_Aeq_) between the UAS and each ambient environment. Figure 1 provides a coarse indication of the prominence of the UAS within each ambient environment and how this relates to annoyance (as discussed further below, there are more accurate approaches to determining how much the UAS sound obtruded from the ambient sound). The results in Fig. 1 indicate that annoyance ratings were associated with UAS *L*_Aeq_, and the association varied between the two environments: annoyance was generally rated lower in the calm urban park environment (CUPenv) than in the busy city street environment (BCSenv), but also shows a steeper association with increasing UAS sound level.

**Fig. 2:**
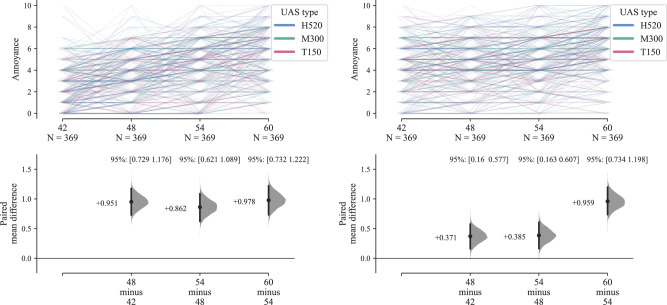
Part A results (*n* = 41): Annoyance effects by UAS *L*_Aeq_ and ambient environment; left, calm urban park; right, busy city street (Cumming estimation plots for within-subjects paired differences, effect size as sequential paired mean difference with 95% confidence intervals from bootstrap sampling over 5000 iterations, line plot data points *xy*-jittered).

The size of the effect of increasing UAS *L*_Aeq_ interval on annoyance ratings has been estimated separately for each ambient environment using a within-subjects paired differences effect size bootstrapped estimation analysis^12^, as shown in Fig. 2. Each Cumming estimation plot comprises two parts: the upper part shows the entirety of the raw data analysed, which in this case is rendered as lines tracing each set of responses for each individual participant across all the corresponding categorical combinations (colours are used to distinguish co-varying factors); the lower part displays the paired mean differences alongside the distributions and (bias-corrected and accelerated) confidence intervals for the differences, obtained using the empirical bootstrap method (NB: The ‘N’ in the effect size estimation plots refers to the number of paired responses, rather than the number of participants, *n*). In this case, the full factorial components of the test comprised 72 stimuli per participant (4 × 3 × 3 × 2), which in Fig. 2 are divided among the 2 ambient environments. This analysis indicates that the effect within the CUPenv (in terms of mean differences for participant-matched observations) yields a consistent elevation of approximately 1× annoyance rating interval per 6 dB *L*_Aeq_ interval step increase (see Supplementary Table 1 for standardised effect sizes). For the BCSenv, the effect size is smaller at low UAS levels (less than 0.5× an annoyance rating interval), but increases to a similar effect size at the highest *L*_Aeq_.

The results shown in Figs. 1 and 2 suggest that differences between the two environments played an important role in annoyance judgements. Firstly, the baseline (‘no UAS’) annoyance tended to be higher in the BCSenv than in the CUPenv. Secondly, the growth in annoyance with increasing UAS *L*_Aeq_ was greater in the CUPenv than in the BCSenv: over the *L*_Aeq_ range, typical annoyance ratings within the CUPenv increased by five intervals on the rating scale compared with two intervals in the BCSenv.

**Fig. 3:**
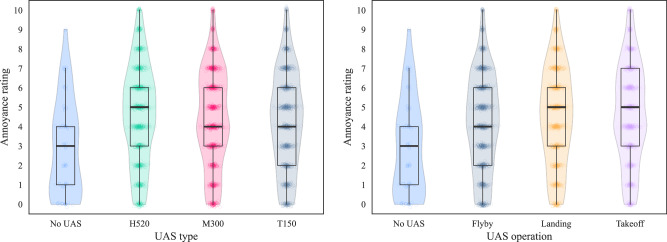
Part A results (*n* = 41): Annoyance ratings; left, UAS type; right, UAS flight operation (violin plots show kernel density with quartile boxes and Tukey interquartile-based whiskers, data points *xy*-jittered).

The results presented in Fig. 3 show the Part A data, segregated by UAS type (Fig. 3 left) and by flight operation mode (Fig. 3 right). The plot in the left of Fig. 3 suggests that the H520 hexacopter was typically associated with slightly higher annoyance ratings than the other UAS types. The plot in the right of Fig. 3 suggests that landing and takeoff operations tended to be rated as slightly more annoying than flyby. Sonic differences between the different types and operating modes that could be relevant to these observations are explored below.

**Fig. 4:**
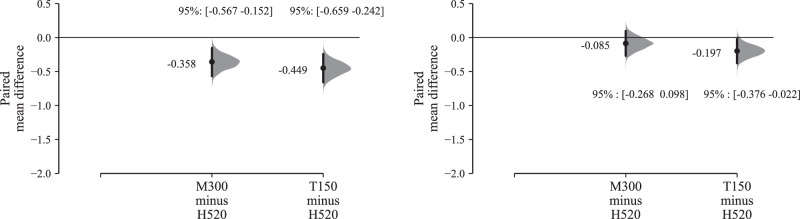
Part A results (*n* = 41): Annoyance effects by UAS type and ambient environment; left, calm urban park; right, busy city street (Cumming estimation plots for within-subjects paired differences, effect size as sequential paired mean difference with 95% confidence intervals from bootstrap sampling over 5000 iterations, line plot data points *xy*-jittered; see Supplementary Fig. 1 for the full data upper plot sections).

The effect size estimation shown in Fig. 4 reveals that, while the H520 hexacopter was typically rated as more annoying than the M300 quadcopter and the T150 X8-copter, this difference was smaller within the BCSenv compared with the CUPenv. Figure 4 also indicates that the overall effect size was relatively slight (on average), at typically less than 0.5× an annoyance rating interval (Cohen’s *d* for T150 vs. H520, park: −0.178 [−0.261, −0.094]; for M300 vs. H520, park: −0.143 [−0.225, −0.060]—see Supplementary Table 2).

Further analysis shows that, when considering only the highest UAS sound level (i.e., 60 dB *L*_Aeq_), the observed effect of UAS type on annoyance judgements grew larger in the CUPenv but not substantively for the BCSenv; for the park the effect rose towards approximately one annoyance rating interval (Cohen’s *d* for T150 vs. H520, park: −0.306 [−0.498, −0.124]; for M300 vs. H520, park: −0.385 [−0.570, −0.198]—Supplementary Table 2), while a negligible change is observed for the street environment (Supplementary Fig. 2).

**Fig. 5:**
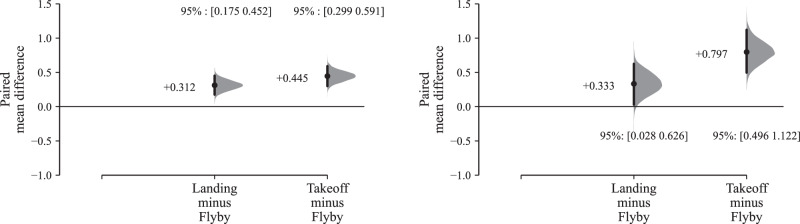
Part A results (*n* = 41): Annoyance effects by UAS operation; left, all UAS *L*_Aeq_ intervals; right, UAS *L*_Aeq_ 60 dB (Cumming estimation plots for within-subjects paired differences, effect size as sequential paired mean difference with 95% confidence intervals from bootstrap sampling over 5000 iterations, line plot data points *xy*-jittered; see Supplementary Fig. 3 for the full data upper plot sections).

A corresponding estimation plot based on UAS flight operation mode is shown in Fig. 5, which indicates that flyby operations were generally assigned lower annoyance ratings compared with either landing or takeoff (for this variable there was no substantive difference in the results segregated by ambient environment, so Fig. 5 presents the combined results; the segregated effects can be viewed in Supplementary Fig. 4). The overall effect sizes were again relatively small, typically less than 0.5× an annoyance rating interval (Cohen’s *d* for takeoff vs. flyby: 0.177 [0.119, 0.236]—see Supplementary Table 3).

Filtering the effects of UAS operations on annoyance by increasing UAS *L*_Aeq_ values produces less consistent results than observed for the effects of UAS type: while the effect size grew slightly for takeoff vs. flyby at UAS *L*_Aeq_ 60 dB (to Cohen’s *d* of 0.329 [0.204, 0.463]—Supplementary Table 3), the effect size for landing vs. flyby remained almost the same between the combined 42–60 dB *L*_Aeq_ results (Fig. 5 left) and those corresponding with the judgements of the 60 dB *L*_Aeq_-only stimuli (Fig. 5 right).

To complement the non-parametric effect size estimation analysis, a more traditional within-subjects (repeated measures) analysis of variance (ANOVA) has also been used to analyse the data. An advantage of this form of analysis is the ability to test and control for the effects of several factors simultaneously while accounting for the within-subjects clustering, albeit it in a limited manner (as noted by Hoffman and Rovine^13^, within-subjects ANOVA represents a form of linear mixed effects model, with a more restrictive covariance structure than more flexible forms of multilevel regression models).

In the first (base) model for Part A, the within-subjects factors comprise UAS *L*_Aeq_, ambient environment, UAS type, and flight operation, with full factorial interactions. The sphericity assumption has been tested, with Greenhouse-Geisser adjustments applied where Mauchly’s test indicates this is likely (*p* < 0.05) to have been violated (quoted values include these adjustments, as indicated). The results of this model are summarised in Table 1.

**Table 1:** Part A results (*n* = 41): Annoyance effects for within-subjects ANOVA (base model)—within-subjects effects summary

	*F*-statistic	Degrees of freedom^a^		*p*-value	Effect size
Factors		factor	residuals		${\eta }_{{{\rm{p}}}}^{2}$
UAS *L*_Aeq_^a^	107.0	1.68	67.01	<0.001	0.728
Ambient environment	22.11	1.00	40.00	<0.001	0.356
UAS *L*_Aeq_ × ambient env.^a^	16.50	2.43	97.18	<0.001	0.292
UAS operation^a^	13.08	1.62	64.64	<0.001	0.246
UAS type	11.34	2.00	80.00	<0.001	0.221
UAS type × operation × ambient env.	3.213	4.00	160.0	0.014	0.074
UAS *L*_Aeq_ × operation × ambient env.^a^	3.086	4.76	190.3	0.012	0.072
UAS *L*_Aeq_ × operation^a^	2.884	4.74	189.7	0.017	0.067
UAS type × ambient env.	2.554	2.00	80.00	0.084	0.060
UAS *L*_Aeq_ × type	2.268	6.00	240.0	0.038	0.054
UAS *L*_Aeq_ × type × operation	1.193	12.00	480.0	0.285	0.029
UAS *L*_Aeq_ × type × ambient env.	1.091	6.00	240.0	0.368	0.027
UAS *L*_Aeq_ × type × operation × ambient env.^a^	1.037	7.96	318.4	0.408	0.025
UAS operation × ambient env.	0.406	2.00	80.00	0.663	0.010
UAS type × operation	0.073	4.00	160.0	0.990	0.002

*UAS* unmanned aircraft system.

^a^Incorporating Greenhouse-Geisser adjustments for sphericity violations.

The results in Table 1 provide further evidence for the within-subjects effects of UAS *L*_Aeq_, type, operation, and ambient environment. Table 1 also suggests that interaction effects had a notable influence on annoyance: UAS *L*_Aeq_ with the ambient environment; UAS type and operation with the ambient environment; UAS *L*_Aeq_ and type with or without operation; UAS *L*_Aeq_ and operation, with or without ambient environment. With the exception of *L*_Aeq_ × ambient environment, the effect sizes of these interactions are relatively small, indicating subtle interrelationships. Two-way interactions are explored in Figs. 4, 5, and in Supplementary Fig. 2. Further analysis of three-way interactions via estimated marginal means^14^ is provided as Supplementary information (Supplementary Figs. 5, 6), which illustrates that these complexities should not be over-interpreted — the three-way interactions appear to be relatively unimportant.

Using the same within-subjects factors as the base model, a further ANOVA was used to examine the potential influence on annoyance ratings of between-subjects factors (see Execution section for more information): sex, AAM attitude, area of residence (AOR) classification, and AOR soundscape character. The corresponding results of this model are summarised in Table 2.

**Table 2:** Part A results (*n* = 41): annoyance effects for within-subjects ANOVA (between-subjects factors model)—between-subjects effects summary

	*F*-statistic	Degrees of freedom		*p*-value	Effect size
Factors		Factor	Residuals		${\eta }_{{{\rm{p}}}}^{2}$
AAM attitude	2.860	3	31	0.053	0.217
AOR soundscape character	2.175	3	31	0.111	0.174
AOR classification	1.003	2	31	0.378	0.061
Sex	0.204	1	31	0.655	0.007

*AAM* advanced air mobility, *AOR* area of residence.

The results in Table 2 suggest that the effects of between-subjects factors were of lesser importance in influencing annoyance ratings. Participant attitude towards AAM had the largest between-subjects effect.

A further ANOVA incorporated between-subjects covariates: age, Positive and Negative Affect Schedule (PANAS) ratings, and noise sensitivity (see Execution section for further details). The corresponding results of this model indicate that these between-subjects covariates were not of great importance in influencing annoyance ratings, and are provided in Supplementary Table 4.

**Fig. 6:**
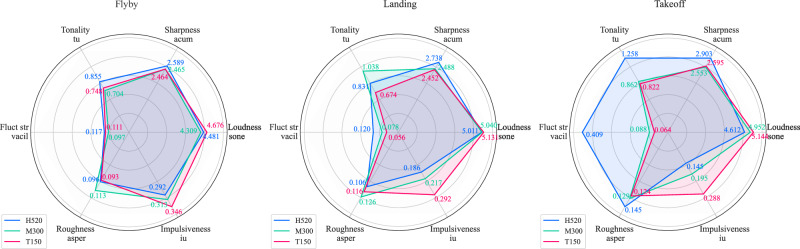
Part A stimuli UAS component sound quality analysis: UAS by operation, for sound level of 60 dB *L*_Aeq_.

Sound quality analysis for a selection of the UAS-only audio renderings used in Part A corresponding with each UAS type and flight operation is shown in Fig. 6 (see ‘Methods’ section for details on metrics and parameters employed).

The analysis in Fig. 6 indicates that:

1. loudness values were similar for all UAS types and operations (as expected, since this analysis is focussed on a single UAS sound level normalisation interval of 60 dB *L*_Aeq_);
2. the H520 hexacopter was sharper than the other UAS in all operating modes, and more tonal in both flyby and takeoff operations;
3. during takeoff, the H520 hexacopter had a relatively high fluctuation strength, and a higher roughness than the other UAS;
4. the M300 quadcopter had higher tonality and roughness values than the other UAS during landing;
5. the T150 X8-copter had higher impulsiveness than the other UAS over all operations.

**Fig. 7:**
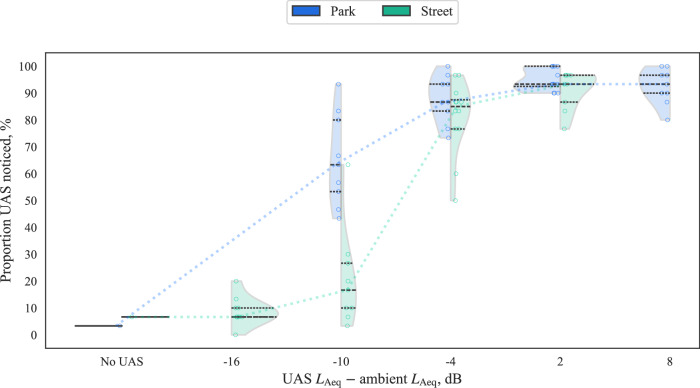
Part A results (participants *n* = 30): Noticeability by UAS ∆*L*_Aeq_ relative to ambient (violin plots show kernel density and black quartile lines, coloured dotted lines track median values, data points *x*-jittered).

The proportions of participants with ‘UAS noticed’ classifications (i.e., ‘noticeability’) for each interval of UAS sound level relative to the ambient sound are shown in Fig. 7. In the BCSenv, noticeability typically remained below 20% up to relative levels of −10 dB ∆*L*_Aeq_, then increased rapidly at the next highest interval (−4 dB) to ~85%. By contrast, in the CUPenv, noticeability was typically >50% at 10 dB ∆*L*_Aeq_, and increased to similar proportions to the BCSenv for higher intervals (but remained typically higher than the BCSenv for each matching interval). Interestingly, the greatest spread in the results is observed for both environments at −10 dB ∆*L*_Aeq_—here, it should be remembered that each interval in Fig. 7 includes all the corresponding variations of UAS type and operation at each sound level, which would explain much of the variation in noticeability; the differing time profiles and sound characteristics of each combination of ambient environment, UAS type and operation would be expected to result in varying noticeability.

**Fig. 8:**
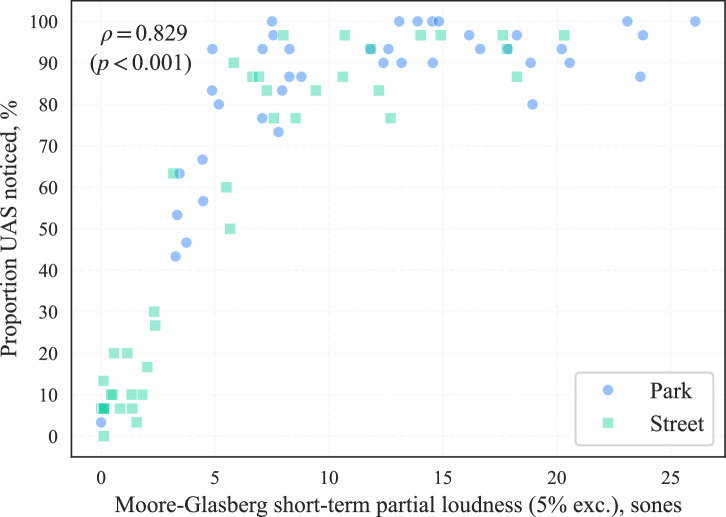
Part A results (participants *n* = 30): Noticeability by partial loudness (Spearman’s *ρ* correlation coefficient for combined data).

A more sophisticated approach to determining the perceived loudness of a time-varying sound potentially masked by another time-varying sound can be found in models for partial loudness^15,16^. Partial loudness represents the residual loudness of the sound component of interest that remains unmasked by the masking sound; in this case, the partial loudness of a UAS after accounting for the masking by the ambient environment. Figure 8 shows the results of analysing the correlation between noticeability and partial loudness (calculated using a Python/C++ implementation of the Moore-Glasberg algorithms^17^ and applying a 95^th^ percentile aggregation over the time-dependent values). This illustrates that the noticeability differences between the ambient environments shown in Fig. 8 can be substantively explained by variations in the corresponding partial loudness; the analysis indicates that a partial loudness of at least ~4 sones appeared to be noticeable to the majority of participants, according to this model for partial loudness.

**Fig. 9:**
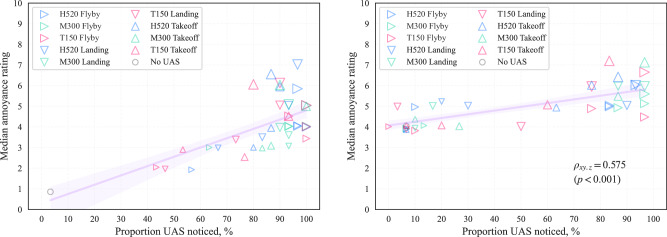
Part A results (participants *n* = 30): Annoyance related to noticeability; left, calm urban park; right, busy city street; (partial Spearman’s *ρ*_*xy*.*z*_ correlation coefficient for combined data, controlling for ambient *L*_Aeq_; linear regression lines with 95% confidence intervals from bootstrap sampling over 5000 iterations; data points *y*-jittered, point size proportional to UAS *L*_Aeq_).

The relationship observed between noticeability and annoyance is illustrated in Fig. 9, which suggests that:

- there are differences in the associations across the contrasting ambient environments, with a steeper growth in annoyance with increasing noticeability observed for the CUPenv;
- controlling for the ambient environment, the correlation of annoyance with noticeability for this set of stimuli is moderately strong;
- the association of annoyance with noticeability within each ambient environment appears to have a convergence tendency.
- Higher ratings of annoyance at similar noticeability tended to be associated with landing or takeoff operational modes, whereas the flyby operations may be equally (or more) noticeable, but were typically less annoying.

**Fig. 10:**
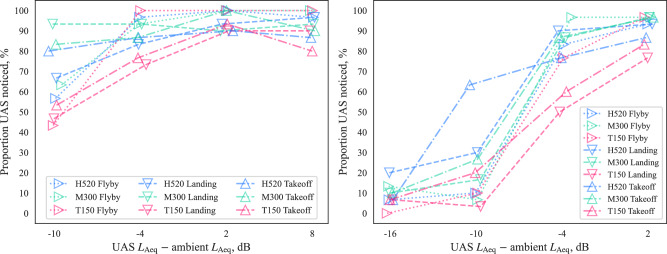
Part A results (participants *n* = 30): Noticeability by UAS Δ*L*_Aeq_ relative to ambient; left, calm urban park; right, busy city street (data points *x*-jittered).

The influence of UAS type and flight operation on noticeability can be further examined in Fig. 10, which indicates that the T150 X8-copter was typically less noticeable than the other UAS types, except during flyby operation. In the BCSenv (Fig. 10 right), the H520 takeoff was much more noticeable at −10 dB ∆*L*_Aeq_ than all other combinations, indicating that its particular sound qualities or time profile were prominently distinctive within the soundscape. In the CUPenv (Fig. 10 left), flyby operations tended to be most noticeable, at least for UAS ∆*L*_Aeq_ exceeding −10 dB; at −10 dB, landing or takeoff operations were typically more noticeable, with the M300 landing approaching 100% noticed.

### Part B

The focus of Part B was the effect of varying the quantity of UAS flyby events, or equivalently, ‘event density’ (normalised by overall stimulus duration). As detailed in the Methods section, this part of the experiment narrowed the selection of UAS types to the H520 hexacopter and the T150 X8-copter, each of which was normalised to two overall sound levels (54 dB and 60 dB *L*_Aeq_), and embedded within the CUPenv only. The normalisation of the UAS sounds was applied over the whole stimulus duration, which means that the sound energy of individual UAS events was reduced proportionately with increasing event quantities. Since the overall UAS *L*_Aeq_ was held constant at each interval throughout the varying event quantities, a steady judgement of annoyance with increasing event quantities would indicate that constant (overall) sound energy corresponded with a constant subjective response. This notion that a balance between individual source event sound energy, event quantities, and duration can be related to a constant response is commonly described as the ‘equal energy principle’ or ‘equal energy hypothesis’^18,19^.

**Fig. 11:**
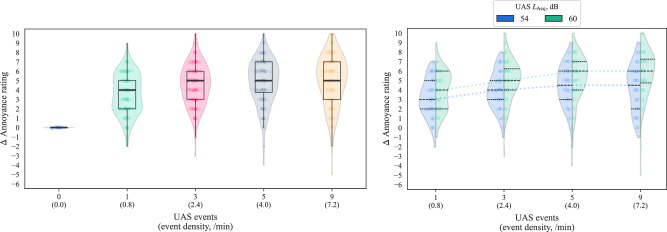
Part B results (*n* = 42): Annoyance ratings by UAS event quantity/density; left, combined data (violin plots show kernel density with quartile boxes and Tukey interquartile-based whiskers, data points *xy*-jittered); right, segregated by UAS *L*_Aeq_ (violin plots show kernel density estimate and black quartile lines, coloured dotted lines track median values, data points *xy*-jittered).

The Part B annoyance results are shown in Fig. 11, which combines the data for both UAS types. The results in Fig. 11 indicate that annoyance ratings tended to increase with both UAS *L*_Aeq_ and quantity of events, but event quantity-related increases in annoyance appear to have ‘saturated’ from around 3–5 events (2.4–4.0/min). This flattening of the responses indicates increasing agreement with the equal energy hypothesis as event quantities are increased.

**Fig. 12:**
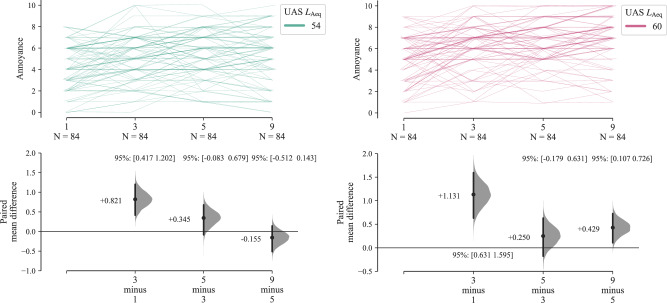
Part B results (*n* = 42): Annoyance effects by UAS event quantity and *L*_Aeq_; left, UAS *L*_Aeq_ 54 dB; right, UAS *L*_Aeq_ 60 dB (Cumming estimation plots for within-subjects paired differences, effect size as sequential paired mean difference with 95% confidence intervals from bootstrap sampling over 5000 iterations, line plot data points *xy*-jittered).

Effect size estimation plots for the Part B results are shown in Fig. 12 as paired sequential comparisons—here, deviation from the 0 difference line can be interpreted as potential evidence against the equal energy hypothesis; a positive difference indicating that the UAS multiple events sequence is typically rated as more annoying that would be expected if equal (A-weighted) energy was a stronger explanatory factor than the numbers of events (and vice versa). Figure 12 shows that the comparison between 3 vs. 1 events exhibits a substantive positive difference of approximately 1 annoyance rating interval (Cohen’s *d* for 60 dB *L*_Aeq_: 0.530 [0.283, 0.772]—see Supplementary Table 5)—the participants judged the increase in UAS event numbers to be more annoying, despite the corresponding reduction in individual UAS event sound energy. Further increases in event numbers exhibited smaller effects, although the increased annoyance at 60 dB *L*_Aeq_ for 9 vs. 5 events was larger than for 54 dB, and remained substantively above zero mean difference (Cohen’s *d* for 9 vs. 5 events, 60 dB *L*_Aeq_: 0.198 [0.050, 0.356]; for 54 dB *L*_Aeq_: −0.067 [−0.216, 0.067]—Supplementary Table 5). Further segregation of the results by UAS types (see Supplementary Figs. 7, 8) indicates that the influence of increasing event numbers on annoyance judgements of the H520 hexacopter differed slightly at each *L*_Aeq_ interval from those corresponding with the T150 X8-copter, the latter approaching the equal energy principle more steadily with increasing event quantities.

Applying within-subjects ANOVA to the experiment data from Part B yields the results summarised in Table 3. The ANOVA indicates that the strongest factor in determining annoyance was the overall UAS *L*_Aeq_ interval, with a secondary effect from event quantity and an interaction of event quantity with *L*_Aeq_; UAS type on the other hand had a marginal or negligible effect.

**Table 3:** Part B results (*n* = 42): Annoyance effects for within-subjects ANOVA (base model)—within-subjects effects summary

	*F*-statistic	Degrees of freedom^1^		*p*-value	Effect size
Factors		Factor	Residuals		${\eta }_{{{\rm{p}}}}^{2}$
UAS *L*_Aeq_	61.92	1.00	41.00	<0.001	0.602
UAS event quantity^a^	16.90	1.58	64.79	<0.001	0.292
UAS type	3.722	1.00	41.00	0.061	0.083
UAS *L*_Aeq_ × event quantity^a^	3.322	2.29	93.97	0.034	0.075
UAS event quantity × type	1.227	3.00	123.0	0.303	0.029
UAS *L*_Aeq_ × type	0.252	1.00	41.00	0.618	0.006
UAS *L*_Aeq_ × event quantity × type	0.158	3.00	123.0	0.924	0.004

*UAS* unmanned aircraft system.

^a^Incorporating Greenhouse-Geisser adjustments for sphericity violations.

Incorporating between-subjects factors into the base ANOVA model produces the results summarised in Table 4. Considering these results alongside the corresponding Part A results in Table 2 suggests that two between-subjects factors appear to warrant further examination: participant attitude towards AAM, and the classification of their AOR. These aspects are addressed in the following section. An ANOVA model incorporating the between-subjects covariates for Part B again indicates very little influence, the results of which are shown in Supplementary Table 6.

**Table 4:** Part B results (*n* = 42): annoyance effects for within-subjects ANOVA (between-subjects factors model)—between-subjects effects summary

	*F*-statistic	Degrees of freedom		*p*-value	Effect size
Factors		Factor	Residuals		${\eta }_{{{\rm{p}}}}^{2}$
AAM attitude	2.682	3	32	0.063	0.201
AOR classification	3.679	2	32	0.036	0.187
AOR soundscape character	2.257	3	32	0.101	0.175
Sex	1.158	1	32	0.290	0.035

*AAM* advanced air mobility, *AOR* area of residence.

### Personal factors

Considering that the AAM attitude distribution (see Fig. 19 in Participants section) has reasonably well-balanced representation among the four response options (‘supportive’ / ‘neutral’ / ‘ambivalent’ / ‘concerned’), the participant sample does not undermine testing for a potential effect of attitude on responses.

**Fig. 13:**
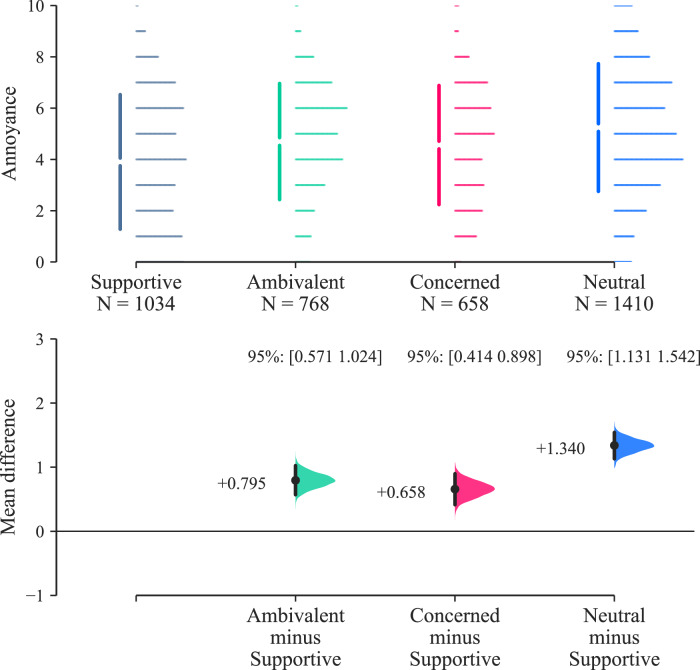
Parts A & B results (*n* = 42): Annoyance effects by participant attitude towards AAM technology (Cumming estimation plot for between-subjects differences, effect size as mean difference with 95% confidence intervals from bootstrap sampling over 5000 iterations).

Effect size estimation analysis of participant AAM attitude is shown in Fig. 13 (which incorporates results from both parts of the experiment). The results in Fig. 13 indicate that participants with a ‘supportive’ attitude towards AAM technology were more likely to respond with lower annoyance ratings than those with other attitudes; the typical differences were between approximately 0.7 and 1.3× an annoyance rating interval (Cohen’s *d* for ‘supportive’ vs. ‘neutral’: 0.526 [0.442, 0.608]—see Supplementary Table 7).

**Fig. 14:**
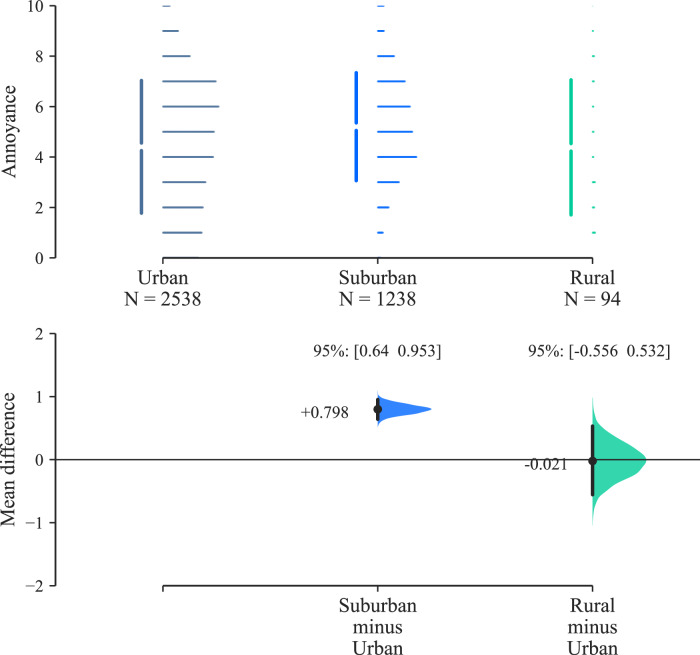
Parts A & B results (*n* = 42): annoyance effects by participant area of residence classification (Cumming estimation plot for between-subjects differences, effect size as mean difference with 95% confidence intervals from bootstrap sampling over 5000 iterations).

Effect size estimation for AOR classification (Fig. 14) indicates that participants classifying their AOR as ‘surburban’ responded with higher annoyance ratings than those from ‘urban’ areas, with a difference of ~0.8× an annoyance rating interval (Cohen’s *d*: 0.322 [0.258, 0.384]—Supplementary Table 7). No inference on effects is drawn from the results in relation to the single ‘rural’-classified participant, due to the lack of sample balance for this group (see Fig. 20).

## Discussion

This study investigated perception and responses to a range of different UAS types, flight operations and event quantities embedded in varying ambient acoustic environments using a within-subjects listening test design. A detailed analysis of the results indicates that all the controlled variables (UAS *L*_Aeq_, design type, flight operational mode, and ambient environment) had a substantive effect on annoyance ratings and (indirectly-derived) source noticeability. Annoyance judgements were also affected by UAS event quantities.

**Fig. 15:**
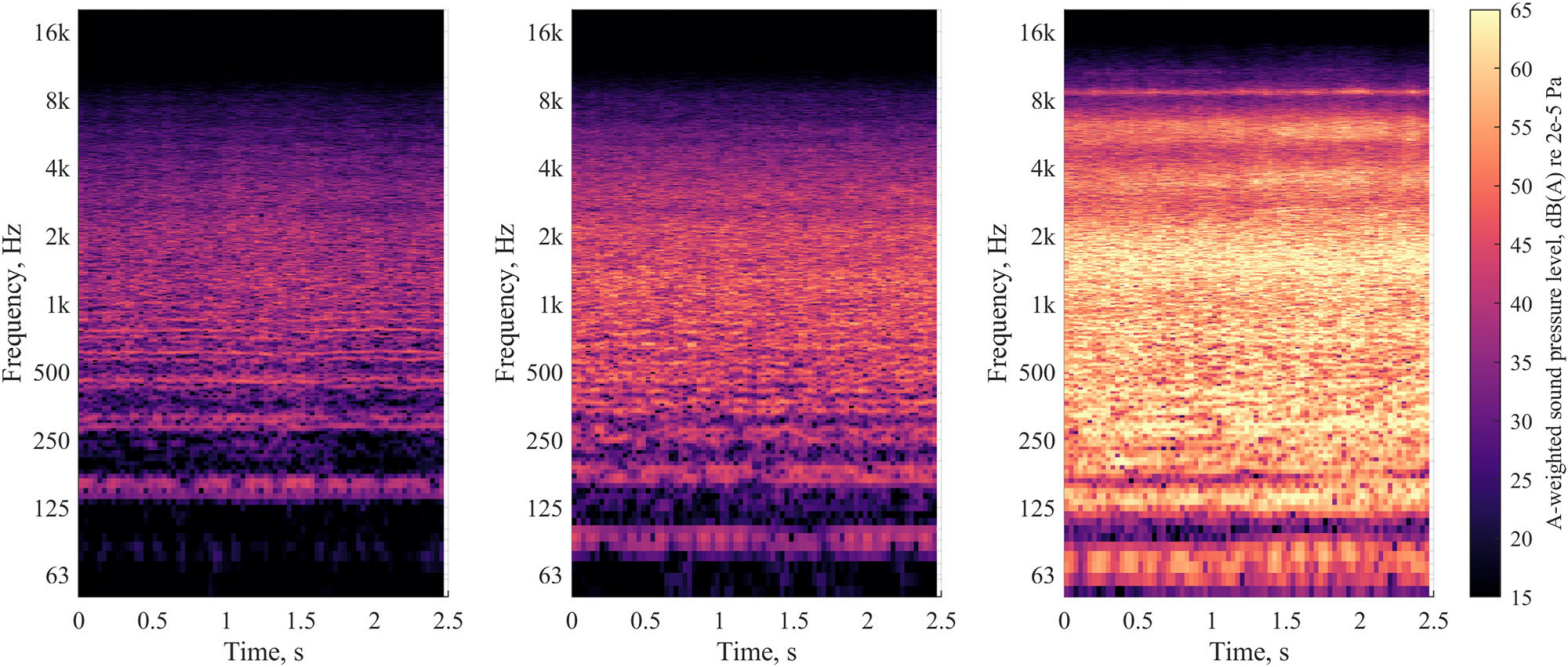
Spectrograms of UAS in hover operation (close-range monaural recordings); left: H520 hexacopter at 10.4 m distance; middle: M300 quadcopter at 10.4 m distance; right: T150 X8-copter at 13.6 m distance (∆*f* 8 Hz, ∆*t* 1/32 s, Hann-windowed 75% overlap).
- The relationship between noticeability and annoyance was evident, and also clearly dependent on the ambient environment: in the BCSenv, generally higher ratings of annoyance did not consistently diminish with decreasing noticeability, and only increased a little at high noticeability. This outcome seems to be an inherent consequence of a more annoying ambient environment, in which the presence or absence of a UAS source becomes less important in determining the overall response than in a less annoying environment. Annoyance appeared to be more influenced by noticeability in the CUPenv, but with a wider variation in responses. The variations appeared to be somewhat associated with the differences in responses to varying UAS flight operations, with flybys tending to be less annoying, despite being similarly or more noticeable than takeoff or landing modes. As UAS noticeability increased in the CUPenv, the association with increasing annoyance appeared to converge with the responses for the BCSenv, which is consistent with the notion that the influence of ambient sound decreases in importance as the prominence of a particular source is elevated.

**Fig. 16:**
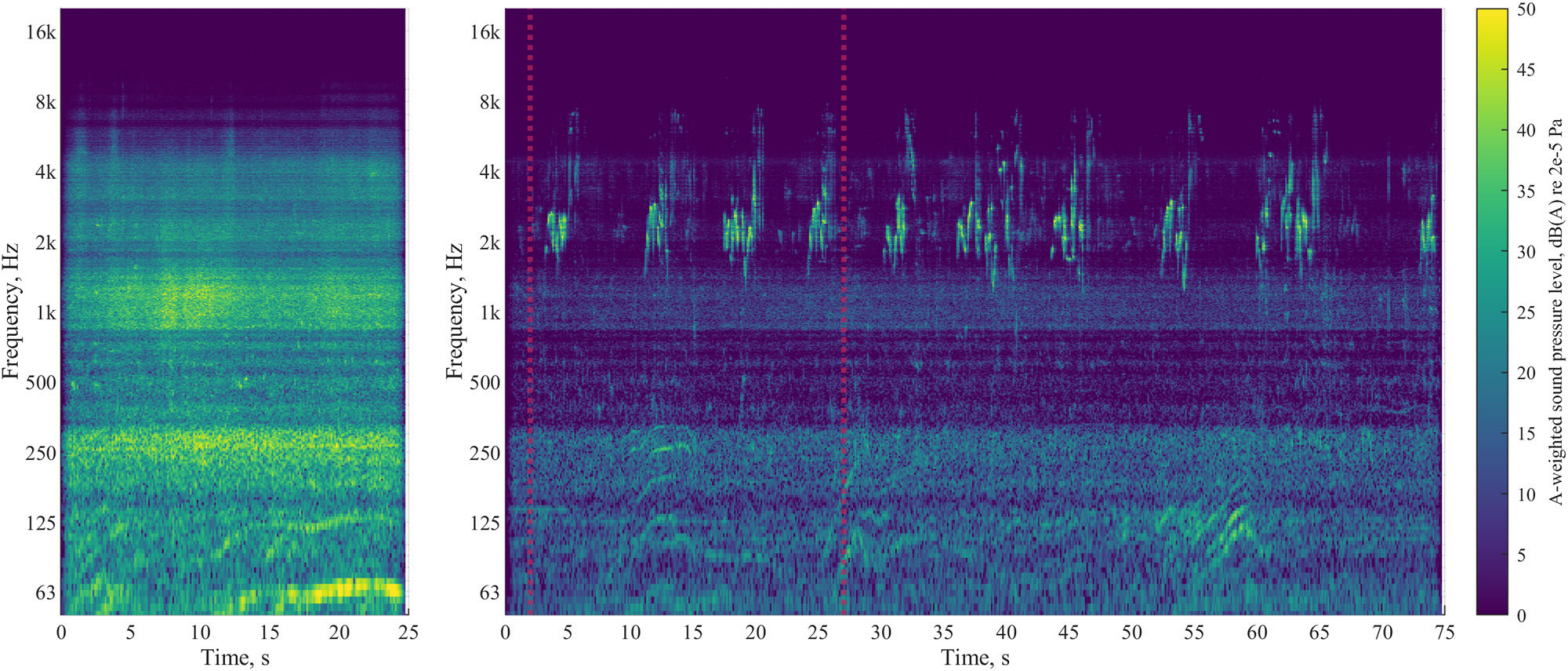
Spectrograms of ambient acoustic environments (binaural recordings at the participant position, incoherently summed); left: busy city street; right: calm urban park—vertical dotted lines indicate section used in Part A of the experiment (∆*f* 4 Hz, ∆*t* 1/8 s, Hann-windowed 50% overlap).

Part A of the experiment was focussed on the effects of varying UAS types, operations and ambient environments, which identified the following:

- Annoyance ratings increased with UAS *L*_Aeq_, and this relationship differed between the two ambient environments: in the CUPenv, the effect was consistent over each increase in *L*_Aeq_ interval; in the BCSenv, initial increases in UAS *L*_Aeq_ interval (42 dB to 48 dB to 54 dB) were associated with smaller increases in annoyance, while a further increase in *L*_Aeq_ to the top interval (60 dB) resulted in a larger effect, commensurate with that for the CUPenv. This indicates that increasing UAS *L*_Aeq_ tended to have a stronger effect in terms of increases in annoyance within the CUPenv, an observation that is consistent with previous UAS research findings by Torija et al.^20^. This evidence supports the notion that UAS sound would have a greater adverse ‘change’ impact in a calmer, quieter environment than a busier, noisier one.
- On the other hand, it was also observed that *absolute* ratings of annoyance tended to be lower in the CUPenv than in the BCSenv. This indicates that, (i) as would be expected, the CUPenv is judged to be a less annoying acoustic environment, and (ii) that the contextual incongruence between a relatively loud UAS source and a quiet, calm urban soundscape did not lead to elevated overall annoyance ratings compared with a busier, noisier environment (the BCSenv). This further suggests that, in terms of absolute annoyance, the restorative benefits of a more pleasant soundscape may have compensated for the noise impact introduced by the UAS. Further work could investigate how judgements are influenced in environments with greater ecological validity, incorporating visual context—for example, experimental research by Aalmoes et al.^21^ identified elevated absolute annoyance for UAS sounds within an immersive audio-visual representation of a quiet, rural environment compared with a busy city street.
- Annoyance ratings were also affected by both UAS type and flight operation:
  - The small H520 hexacopter tended to be rated as more annoying that either the medium M300 quadcopter or the large T150 X8-copter, and this effect was stronger at higher UAS *L*_Aeq_. The effect of UAS type was smaller in the BCSenv than in the CUPenv. This result indicates that the differences in UAS characteristics were more distinguishable in the quieter CUPenv, within which UAS sound characteristics may become more important in subjective judgements.
  - Flyby operations were rated less annoying than either landing or takeoff, with takeoff tending to be rated the most annoying operation. Experimental work by Green et al.^9^ identified a similar result for a different range of UAS models, observing that flybys were rated least annoying, but with landing rated more annoying than takeoff. However, their initial observation was tempered by the recognition that differences in sound levels between the recorded operations could have influenced the results. In further analysis using an augmented (dummy variable) linear regression ‘offset analysis’, Green et al.^9^ identified that offsets required to equalise modelled annoyance to a flyby baseline were very similar between takeoff and landing, suggesting that the perceived loudness differences between these operations could have been influencing the observed difference in annoyance. By contrast, in the present study, sound levels were normalised across stimuli groups to control for loudness differences.
  - Sound quality analysis of each of the nine combinations of UAS type and operation tested suggests the possibility that sound qualities (in particular, tonality) associated with landing and takeoff operations could be a potential factor influencing judgements, while the elevated annoyance associated with the H520 hexacopter could also be related to consistently higher tonality and sharpness. Moreover, there are apparently complicated sound quality interactions between the various UAS types and flight operations that may help to explain why the observed effect sizes appear to be relatively slight when tested on the overall data, as well as the influential interactions identified in Table 1. Overall, the most annoying combination of UAS type and operational mode was the H520 in takeoff, which also corresponded with elevated tonality, sharpness, roughness, and fluctuation strength (Fig. 6). The expectation that sound qualities influenced annoyance judgements is also consistent with several previous UAS exposure-response studies^5,22^.
  - Our results for UAS operational mode and vehicle type are consistent with those of Kawai et al.^23^, who observed that a smaller quadcopter (0.9 kg) was judged as more annoying than a larger quadcopter (6.3 kg), with landing and takeoff operations being more annoying than flybys at equal sound levels. Our data indicate these findings encompass a wider range of UAS sizes and configurations, from a much larger (60 kg), contra-rotor X8-copter, to a small (2 kg) hexacopter.
- Noticeability of the UAS was consistently higher in the CUPenv over all sound levels, as expected. A strong correlation between noticeability and partial loudness was observed, irrespective of the ambient environment. This suggests that partial loudness could be a good predictor for UAS noticeability. Further investigations could consider whether partial loudness models, or other, less computationally costly source detection metrics (for example,^24^), are useful in predicting noticeability (or annoyance).
- Noticeability also appeared to be influenced by UAS type and flight operation, with flyby operations tending to be most noticeable in the CUPenv at higher sound levels while landing and takeoffs were more noticeable at the lowest *L*_Aeq_. This could be related to the greater spatial variation in the flyby events, as well as differences in the loudness time profile compared with takeoff or landing operations. The lower-frequency, more broadband T150 X8-copter tended to be less noticeable than the other UAS types, which could be due to sound masking effects associated with spectral overlap from road traffic sound (Figs. 15, 16).

Part B of the experiment focussed on the effects of multiple UAS flyby events, making the following observations:

- Annoyance ratings tended to initially increase with numbers of UAS events, but this effect was consistent only for the increase between 1 and 3 events (0.8–2.4/min).
- For further increasing quantities (2.4–4.0–7.2/min), the sequential effects of event quantity varied slightly depending on the overall *L*_Aeq_ interval: at the higher interval (60 dB), the influence of event quantity appeared to be slightly stronger than for the lower interval (54 dB), suggesting that the higher prominence of the sound events within the ambient exacerbated the impact of UAS event repetition.
- The influence of UAS type was considerably less important than in the single event (Part A) test: Part B responses to the T150 X8-copter had a slightly stronger tendency towards the equal energy hypothesis for more numerous events, while annoyance ratings of the H520 hexacopter tended to be slightly more affected by increasing event quantities. However, the ANOVA indicated that UAS type had no substantive effect. This suggests that, for multiple event scenarios, the importance of other sound qualities (influenced by UAS type) diminished in comparison with loudness and the event repetition. Another factor could have been the Part B focus on flyby events, which would have made the sounds in Part B more homogeneous than the more varied flight operations tested in Part A (see Fig. 6). Future work on multiple UAS events could consider whether non-periodic sequences and other operational modes elicit different effects to the regular patterns of flybys employed here.

The outcomes from analysis of personal (between-subjects) factors indicate that participant AOR classifications and attitudes towards AAM technology had some influence on annoyance ratings:

- With regards to attitude, this result is consistent with previous experimental UAS research, which found that a pre-existing negative attitude towards UAS was associated with higher annoyance responses to audio-visual scenes of UAS in urban environments than a more positive attitude^7^. This further supports the natural intuition that more positive views on AAM technology would be associated with decreased UAS noise annoyance. This observation also highlights the potential value in effective communication and engagement with communities affected by future UAS deployments.
- In relation to AOR, the data suggest that suburban residents tended to be more annoyed than urban residents. Similar influences have been identified previously in observational studies of environmental noise (for example,^25^), however in relation to experimental exposure to UAS sound, this indicates a potentially novel result, which could warrant further research. Experimental work with UAS sounds has indicated geographic regional effects on responses^8^, but did not examine the stratification of regions into area classes.

The other personal factors investigated here did not appear to have a substantive effect on responses. The apparent lack of an observed effect of noise sensitivity is consistent with previous experimental UAS research^7,26^. However, in observational studies of other sources of environmental sound (including conventional aircraft), noise sensitivity has been identified as an important influencing factor for noise annoyance^27,28^. These observations suggest that individual noise sensitivity is more important for long-term (chronic) noise annoyance, usually obtained in social survey recall-based questionnaires, compared with the short-term (acute), momentary noise annoyance typically examined in controlled experiments.

It is recognised that a within-subjects ANOVA has limitations as a multilevel modelling technique for repeated measures experiment designs. Its use here is primarily focussed on testing for potential effects of personal factors on annoyance, with a view to identifying variables of potential interest for further investigation. One limitation of the ANOVA approach is that interactions for between-subjects variables can only be tested (i.e., modelled) if that combination is represented at every level of each within-subjects factorial combination — for example, in this case, this precludes testing whether there are influential interactions between AOR classification and AAM attitude. Having identified variables of potential interest, further research could involve use of regression modelling to evaluate the importance of more complex interactions, as well as to analyse the potential importance of trial ordering effects.

The study had an unbalanced female:male ratio, which limits the ability to draw conclusions on this aspect. However, previous experimental listening studies have indicated that this factor is commonly not an important influencing factor in laboratory tests for noise annoyance outcomes (for example,^29,30^). Nearly two-thirds of the participant sample comprised urban residents, with only one participant from a rural area. This representation is unsurprising, given the recruitment and location of the experiment (see ‘Methods’ section), but implies caution in relation to analysis of AOR classification in this case. Further research could attempt to broaden the representation, for example by use of a remote (online) experimental setup, at the probable cost of reduced immersion and consistency in exposures.

Concerning ecological validity of the experiment, it is recognised that audio-only exposures are less realistic than audio-visual and virtual reality approaches, which means that there will be differences compared with judgements obtained in more realistic environments^31^. On the other hand however, there are some advantages to excluding visual components—this approach implies that differences observed between the ambient environments and between the different UAS types or operations are due to aural qualities, and not to visual differences, which is more difficult to establish within audio-visual setups. Having established a greater understanding of the influence of aural factors, a natural progression in this study will be to investigate the effects of incorporating visual contexts for the environments and UAS, to increase ecological validity and enhance the contextual basis for stimuli evaluation.

In relation to the study research questions and hypotheses, the following conclusions can be drawn:

- The results of this experiment show that, first and foremost, varying UAS *L*_Aeq_ had a strong effect on noticeability and annoyance, confirming our hypothesis. The data also suggest that tonality and sharpness had an influence on judgements, which would be consistent with some previous results^5^. Further investigation is needed to more firmly establish the relative importance of the various sound qualities, which are clearly interrelated with varying UAS type and operation.
  - The experiment shows that the smaller hexacopter was typically judged as more annoying than the other types tested (a medium-sized quadcopter and a large X8-copter), and also tended to be more noticeable when in flyby operation within the quieter, calm urban park ambient environment.
  - Sound quality analysis and inspection of spectrograms for the hexacopter (see Stimuli section) show that the sound has prominent tonal energy, which is also concentrated towards the mid-frequency spectral region (which subjectively manifests as a ‘buzzing’ timbre).
  - Flyby operations were generally found to be less annoying that landing and takeoff operations (confirming our hypothesis), despite being sometimes equally or more noticeable—this suggests that the more uniform transitory nature of flybys may impart an inherently reduced adverse impact compared with other modes.
- The effect of UAS loudness on annoyance was much clearer within the calm urban park environment than within the busy city street: increasing sound levels were strongly associated with growing annoyance. By contrast, in the busy city street, annoyance was generally higher at any UAS sound level, and the contribution of the UAS to increasing annoyance was correspondingly smaller than in the park environment. From this, it appears that our hypothesis of greater annoyance associated with UAS sounds in calmer, quieter environments was too simplistic: the more pleasant environment appears to have compensated (to an extent) for the UAS sound in terms of the overall response, although annoyance grew at a faster rate in the park with increasing UAS *L*_Aeq_. For noticeability, the noisier BCSenv clearly provided more effective masking for the UAS sounds, with greater noticeability in the CUPenv observed at equal UAS sound levels. Moreover, noticeability had a steeper association with annoyance in the CUPenv. This evidence therefore supports the suggestion that routing UAS flight paths near to existing transport routes could be a useful strategy to mitigate *increases* in noise annoyance^20^. On the other hand, if the optimisation constraint objective is to minimise *absolute* noise annoyance, the evidence from this study suggests that such a strategy would only be advisable if the UAS were operated at a sufficient altitude to avoid increasing sound levels (exacerbating adverse impacts) in noisy areas further.
- The associations observed between noticeability and annoyance varied with UAS operational mode—landing and takeoff operations had a greater adverse impact when highly noticeable, whereas flybys could be equally noticeable, but rated less annoying. Again, it seems our hypothesis was shown to be somewhat too simplistic on this aspect. It was also observed that noticeability became less influential on annoyance as the UAS sound level increased, as could be expected—this was evident from the convergence of the relationships for each of the ambient environments. Further work is planned to investigate whether these observations persist in more realistic simulations.
- Our results show that increasing UAS numbers (maintaining constant overall energy) initially led to elevated annoyance, but this tended to saturate towards the equal energy principle, supporting our hypothesis. The point of saturation varied with UAS type; event quantities appeared to be slightly more important for responses to the smaller hexacopter than the larger X8-copter.
- Finally, participants who were more supportive of AAM technology tended to be less annoyed by the sounds, while suburban residents were found to be more annoyed than those residing in urban areas. Both of these observations have intuitive potential explanations: people living in urban areas tend to have greater exposure to acoustic environments comprising aircraft or other transportation sounds, and may therefore be more consciously tolerant towards it. Similarly, people feeling greater general support for a technology may also be more disposed to tolerate some aspects that could otherwise be more annoying, such as environmental sound. The analyses presented here do not attempt to examine whether there is interaction between these two factors, which could be the subject of future investigation.

Our findings demonstrate that perception and responses to UAS sound within contextual acoustic environments involve complex considerations and are not simply explained by the vehicle sound level alone. There are several novel aspects of this study that extend the existing evidence base:

- This is the first study to demonstrate effects of multiple UAS flight events on noise annoyance. To the best of our knowledge, only one other study had previously investigated multiple UAS events in relation to affective responses, using audio-visual simulations of multiple UAS in an urban scene^32^. However, noise annoyance was not an outcome measured for the UAS stimuli, and furthermore, the majority of participants did not hear the UAS during the simulations (presumably due to the selection of simulation parameters), whereas in the present study, the UAS remained audible throughout the multiple events part of the experiment.
- This study identified perception and response for a range of UAS that includes (to the best of our knowledge) a considerably larger vehicle than has previously been investigated in UAS studies. Analysis demonstrates that the sound emitted by this larger vehicle is different to the smaller UAS investigated, and corresponding judgements also differed; in this case, the largest UAS (which had a wider broadband, somewhat low-frequency skewed spectrum, featuring less prominent tones in the more-sensitive spectral regions) also tended to be least noticeable and annoying. This is notable, since larger UAS have greater load capacity, which would be important in relation to future expectations for widespread deployment of commercial logistics use.
- No previous study had identified an effect of participant area of residence classification on annoyance responses to UAS sound. One previous relevant study examined broad geographic orientation (west/east) but not land type^8^. It is plausible that residential area classes can contribute to shaping individual expectations relating to noise from environmental sources.

These observations of course should be tempered by acknowledgement of the main, inherent limitations of experimental research: artificial environments with limited samples of participants; such studies call for further confirmation, replication and reproduction in wider settings and samples. Nevertheless, this research advances the understanding of the various factors shaping subjective judgements, aiming to develop effective psychoacoustic models for predicting perception and response to UAS sound within realistic contexts.

## Methods

The experiment comprised two parts, each focussing on different aspects of the research questions: Part A addressed varying UAS types, flight operations and ambient acoustic environments, with judgements of annoyance and an indirect method of identifying noticeability (described in the following sections). Part B focussed on responses to multiple UAS flyby events.

### Stimuli

UAS sound source recordings were obtained from measurement surveys undertaken in the UK and reported previously by Ramos-Romero et al.^33^. The recordings comprised monophonic signals obtained from ground-plate-mounted microphones at close range to each source, with minimal contributions from wind or other extraneous sound sources. Recordings for three different types of UAS were used, spanning a range of sizes, weights, and rotor designs (Table 5).

**Table 5:** Features of UAS used in experiment stimuli

Aircraft parameter	Yuneec H520E (‘H520’)	DJI Matrice 300 (‘M300’)	Malloy T150 (‘T150’)
Rotor design	Hexacopter	Quadcopter	X8-copter^1^
Mass, kg	2	6	60
Size (rotor–rotor span), m	0.5	0.9	2.7

^1^ Contra-rotating octocopter with four twin-rotor supporting arms.

Flight operations captured within the recordings included takeoff, hover, flyby and landing. Spectrograms of the hover recordings (Fig. 15) indicate a range of sound qualities exhibited by the different UAS types. For example, the H520 hexacopter (Fig. 15 left) sound is characterised by prominent, distinguishable blade-pass harmonic tones, which are related to a fundamental around 150 Hz. By contrast, the T150 X8-copter (Fig. 15 right) has a lower fundamental blade-pass frequency ~70 Hz, increased low-frequency and broadband energy, as well as a visible high-frequency tone ~8.6 kHz (thought to be associated with electromagnetic forces in the motors). The M300 quadcopter (Fig. 15 middle) spectrogram indicates a sonic character somewhere in between the H520 and the T150, with a fundamental blade-pass frequency of ~85 Hz, a few somewhat prominent harmonics, and broadband energy less intense than the T150 but more intense than the H520.

The recordings of hover operations were used in an auralisation process based on the technique developed by Heutschi et al.^34^ to generate simulated flyby operations. The auralised flybys incorporated unsteady rotor behaviour, sound attenuation due to geometric distance and atmospheric absorption/scattering, spectral ground effects, and Doppler frequency shifts due to source motion. These simulations were auditioned alongside the flyby recordings by expert listeners (the authors), with satisfactory representation achieved via aural comparisons. For landing and takeoff operations, the close-range recordings were used directly, but with the same sound propagation processing applied. The auralisation and sound propagation modelling approach is presented in further detail by Ramos-Romero et al.^35^ as implemented by Green^36^.

The propagation parameters were selected according to a set of flight paths designed to achieve attenuation (relative to the original close-range recordings) corresponding with a pre-selected set of energy time-averaged sound levels. The flight paths for flyby operations were designed such that the simulation moved the aircraft along a straight line from left–right or right–left, directly in front of the ‘listening position’, at varying perpendicular range and elevation. Flight paths for takeoff and landing were designed to have the aircraft centrally positioned in front of the ‘listening position’, moving along a vertical ascent/descent path and departing/approaching along a fixed 45° angle; the distance to the takeoff/landing point was varied to achieve the desired sound level. Using this approach, a set of source audio renderings were generated for each part of the experiment based on a full factorial design, according to the following parameters:

- For Part A, the stimuli length was set at 25 seconds to accommodate a single UAS event. The UAS sound levels were defined at uniformly spread intervals: 42 dB, 48 dB, 54 dB and 60 dB *L*_Aeq,25s_; these intervals were selected in consideration of the ambient acoustic environments, detailed below. Each of three flight operations (flyby, landing, takeoff) was generated, corresponding with the three UAS types at all four sound levels. An additional six stimuli were generated that applied simple scaling to the UAS flyby events at the highest sound level interval for both environments; this was intended for use as a check on the influence of the geometric flight path variations on subjective judgements in comparison with the corresponding variations in loudness.
- For Part B, the stimuli length was set at 75 s to accommodate multiple UAS events, which comprised sequential flyby operations with event quantities of 1, 3, 5 or 9 per stimulus, sequenced in a regular periodic pattern. The highest two UAS sound levels were used, i.e., 54 dB and 60 dB *L*_Aeq,75s_, with two UAS types, the H520 and the T150—these were selected as representing the extrema of the range of UAS types considered in the experiment (in terms of size and sound qualities, as described above). To maintain these constant UAS time-averaged sound level intervals, the overall UAS component of each stimulus was adjusted according to the number of events by scaling the output level directly (i.e., the geometric modelling parameters applied for each multiple events case was maintained from the corresponding single event).

The ambient acoustic environment sounds were obtained from a database of 1st-order ambisonics recordings^37^. Two contrasting urban locations were selected: (i) a ‘busy city street’ environment (BCSenv) with continuous nearby road traffic and intermittent pedestrian activity; (ii) a ‘calm urban park’ environment (CUPenv) dominated by birdsong and leisure sounds (e.g., faint human conversations), with distant road traffic sound. These selections were made to represent real environments encountered within urban areas, while achieving a distinct difference in sound sources and context. The sections of the recordings used were auditioned and selected to avoid including prominent or alarming events, such as car horns, intelligible speech, shouting, etc. The aim was to ensure that the ambient environments would sound realistic and representative of the associated situational contexts, while being relatively steady and consistent for the duration of the stimulus. Both of the selected recording sections also avoided any audible aircraft movements. Spectrograms of the ambient environment sounds, obtained from binaural recordings using a calibrated head-and-torso simulator (HATS) at the experiment participant position, are shown in Fig. 16.

The two environments were both used in Part A, with the BCSenv presented at 58 dB *L*_Aeq,25s_, and the CUPenv presented at 52 dB *L*_Aeq,25s_. For Part B, a single ambient environment was used, comprising a longer section of the CUPenv recording, presented at the same time-averaged level as in Part A.

For both parts, the ambient environments were combined with each of the UAS source variations, with additional stimuli representing each ‘ambient environment only’ scenario. Example spectrograms of the combined stimuli for each part of the experiment are shown in Supplementary Figs. 9 and 10, derived from the binaural HATS recordings.

**Fig. 17:**
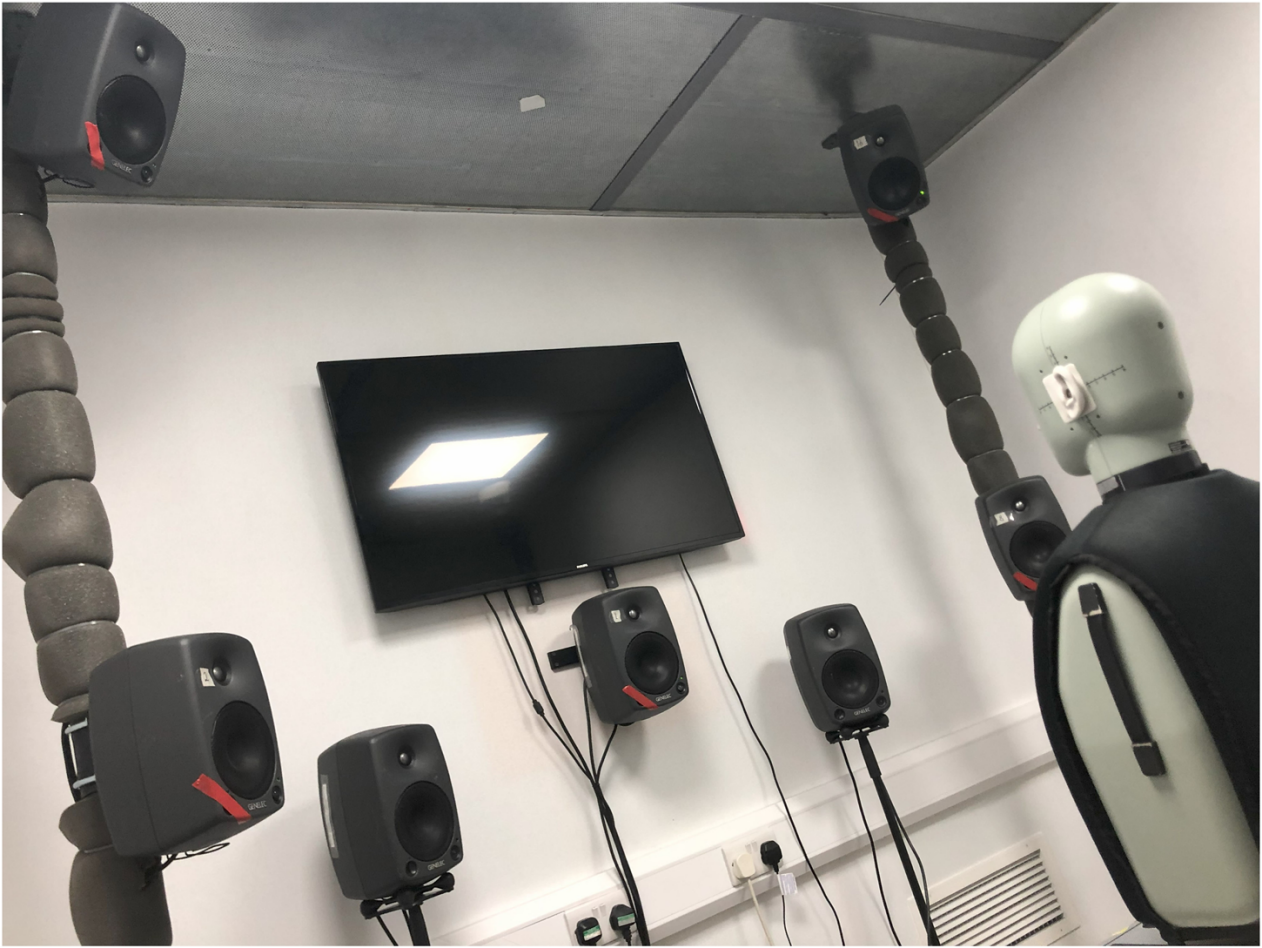
Photograph of test environment (with HATS).

The UAS source and ambient acoustic environment sounds were rendered as 16-channel normalised audio. These audio renderings were then reproduced over a 16-channel loudspeaker system inside a purpose-built, acoustically-treated listening booth (Fig. 17), which has a steady ambient sound level of ≤25 dB *L*_Aeq_ (including contributions from ventilation and all electrical equipment in use), and a reverberation time of around 0.1 s. The presentation included compensation for the specific audio hardware spatial arrangement, which comprised 16 × Genelec 8030A powered loudspeakers positioned in a cuboid array, with 8 × loudspeakers evenly distributed around the azimuthal plane at seated head height, and a further 8 loudspeakers at the positively and negatively elevated corners of the cuboid. The loudspeaker array was configured to produce a consistent (±0.5 dB) sound level (*L*_Aeq_) for a pink noise signal at the central listening position. The loudspeakers were driven from an RME MADIface XT digital audio interface, connected to a Windows 10 computer using MATLAB (2023a) software to trigger playback.

The normalised audio renderings were played over the loudspeaker system and recorded using a Brüel & Kjær type 4100 HATS, connected via a Zoom H4n Pro digital audio interface to a Windows 11 computer using Audacity (3.3.3) recording software. The binaural recordings were used to assign level adjustment factors to each audio rendering to achieve the desired presentation sound levels. The adjusted renderings were then played back and recorded using the HATS setup again, and these binaural recordings then formed the basis for audio analysis. The recordings included both the combined and separated (UAS only) renderings, so that signal analysis could also be undertaken for the UAS components of each stimulus separately.

**Fig. 18:**
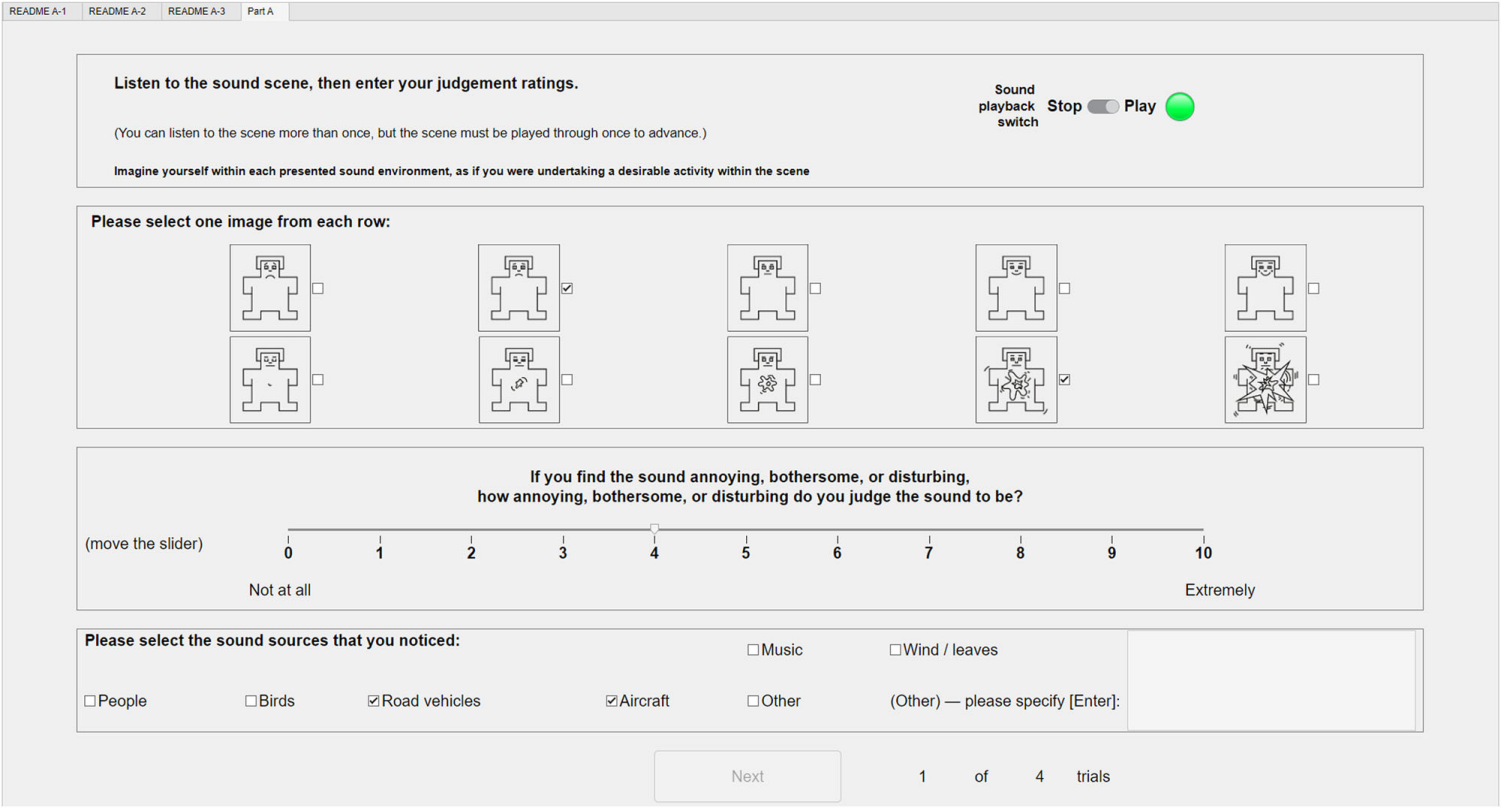
Example of the graphical user interface used by participants in the experiment (example shows Part A practice round page).

Stimulus presentation within each part was randomised, with playback triggering controlled using a graphical user interface (GUI) implemented in MATLAB (Fig. 18). Participants could start, stop and restart each stimulus as desired, but progression through the test required each stimulus to play through to completion at least once. The GUI was presented over a display in the test room. A keyboard and mouse control were provided for participants to use on a small table, which had a ~200 mm thick layer of sound absorption on the remaining surface to mitigate reflections. The computer running the experiment was located in a control area isolated from the booth and with acoustically-treated equipment connection routings.

### Execution

The experiment was granted ethics approval (ID# 14321) by the University of Salford School of Science, Engineering & Environment Ethics Approval Committee, and participants provided informed consent prior to participation.

The two parts of the experiment were both preceded by a briefing section incorporated into the interface, which comprised test instructions and a practice round to familiarise participants with the controls and sounds they would experience. The practice round stimuli were selected from the test set to broadly represent the range of each of the variables under control. Participants could take a short break of up to ~10 min between the two parts of the experiment session, which lasted approximately 60–90 minutes in total.

Prior to the start of the Part A briefing section, participants were asked to declare if they had any known hearing impairments, and to rate their emotional state using the Positive and Negative Affect Schedule (PANAS) scales^38^.

Following the end of the Part B test, participants were asked to enter information concerning their noise sensitivity, experience with and attitude towards AAM technology, demographic details, characteristics of their area of residence, and feedback on the experiment.

The noise sensitivity questions followed the Shortened Form of the Weinstein Noise Sensitivity Scale (SF-WNSS)^39^, with the question ordering randomised for each participant

A multiple-choice question was asked to obtain information on participants’ attitude towards AAM technology (‘supportive’/‘neutral’/‘ambivalent’/‘concerned’).

Multiple-choice questions were also used to ask about the classification of participants’ area of residence (‘rural’/‘surburban’/‘urban’) and the soundscape character of their AOR, as typically experienced (‘calm’/‘chaotic’/‘monotonous’/‘vibrant’—descriptors corresponding with the rotated axes of the ISO 12913-3 soundscape circumplex^40^).

Due to an equipment failure, the Part A test data for 1 participant were not retained (i.e. 41 sets of data were obtained for Part A); the Part B data for all 42 participants were successfully collected.

The test was executed as a within-subjects experiment, with all participants exposed to all combinations of the experimental variables: 80 test stimuli in Part A, and 17 test stimuli in Part B. As noted above, the order of stimuli presentation was randomised for each participant, however, the session order (Part A followed by B) was the same for each participant.

### Participants

Participants were recruited via multiple means: (i) a contact list of previous participants in acoustical listening studies; (ii) flyers were posted around the University of Salford campus, and at nearby leisure facilities; (iii) electronic flyers were advertised via social media and workspace internet applications; (iv) professional networks were also notified via email. The participant information provided to potential recruits advised the requirement for minimum age (18 years) and directed against participation with any known significant hearing impairments. The information also intentionally avoided explicitly mentioning that UAS would be part of the experiment, in an attempt to reduce potential for expectation bias or participation bias—the more general phrase ‘perception of environmental sounds and novel aircraft’ was used to describe the study in recruitment materials. Of 50 initial volunteers, *n* = 42 participants took part in the experiment (drop-out rate of 16%). Participants were provided with financial compensation for their time.

The planned size of the sample was determined based on consideration of previous comparable studies^6,20,41,42^, resource and programme constraints, as well as research indicating that sample sizes for within-subjects experiment designs investigating noise annoyance exhibit ‘diminishing returns’ (in terms of analysis benefit) for samples larger than ~30^43^. Considering the sample size achieved, power analysis for two-tailed paired samples mean difference tests with 328 matched responses (Part A simultaneous testing for effects of UAS type and operation) yields a sensitivity (minimum detectable effect size) of 0.155 (*d*_*z*_) at 80% power up to 0.200 at 95% power. A corresponding analysis for 168 matched responses (Part B testing for effect of flight event quantity) yields values of 0.217 (*d*_*z*_) at 80% power up to 0.279 at 95% power. The smallest substantive standardised effect size noted in the Results analysis was 0.177 (Cohen’s *d*), which corresponded with a change in annoyance of 0.5× a rating interval. Obtaining a larger sample size would increase the likelihood of the detection of smaller effects sizes, but it is unlikely such effects would be of investigative interest. Accordingly, the sample size achieved is considered suitable for the study intent.

Of the 41 participants that confirmed their age, the median age was 31 years, arithmetic mean 33, standard deviation 9 and the age range was 19–58 years. The female:male ratio was 8:34. Of the 42 participants, 16 were University of Salford students, 9 were University of Salford staff, with the remaining 17 of external affiliation.

Participant nationalities spanned a range of geographical regions: of the 39 participants who provided their nationality, just under half were UK nationals. In terms of native languages, the ratio of native English-speakers to other languages was 2:1.

**Fig. 19:**
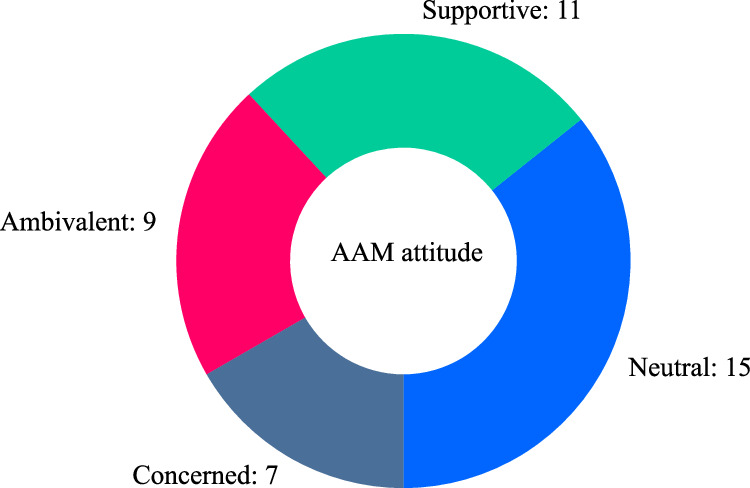
Participant sample attitudes towards AAM technology.

**Fig. 20:**
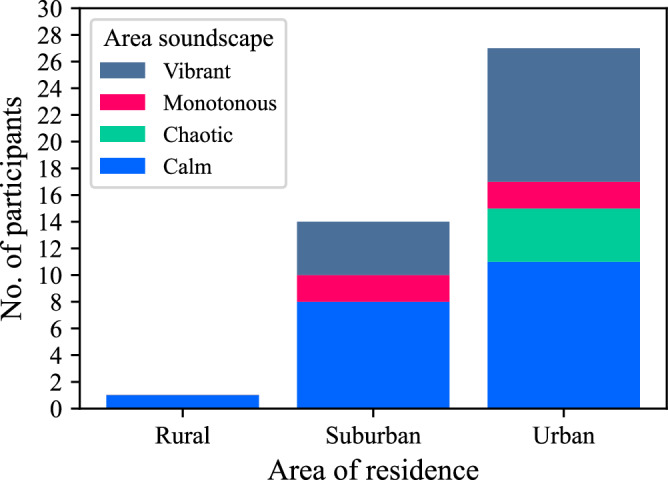
Participant sample AOR classification and typical soundscape description.

Participant responses to the question asked on attitudes towards AAM are summarised in Fig. 19, while participant AOR classifications and descriptions of typical soundscape associated with AOR are shown in Fig. 20.

Participants were instructed to provide three types of judgement in Part A of the experiment. They were also instructed to consider their judgements within the specified context of undertaking a desirable activity within each scene (Fig. 18), and the briefing section of the GUI provided some examples to aid this mental exercise: taking a walk, relaxing, thinking, reading a book, sightseeing, etc. (these examples were specifically selected to imply imagining a ‘quiet’ activity, while also being reasonably plausible across the two ambient environments).

The first judgement comprised affective responses based on the ‘self-assessment manikin’ (SAM, see Fig. 18), which is a pictorial scale representing varying emotions using a simple humanoid-like figure^44^. Various versions of the SAM scale have been used in aural and visual experiments, including soundscape studies^45^. Of the three original emotional components of the SAM, i.e., valence (or pleasure), arousal (or stimulation), and dominance (or control), two components, valence and arousal, have been shown to correspond well with lengthier semantic differential pairs^46^, and also align well with the two axes of the soundscape circumplex model^40,47^. The third component, dominance, has been shown to be of lesser importance in soundscape assessment^46^, and as such has sometimes been omitted^48^. In this experiment, a two-component (valence and arousal), five-interval SAM was used, as shown in Fig. 18.

A second form of affective judgement comprised the ISO/TS 15666^49^ annoyance rating defined as an 11-point (0–10) discrete numerical scale with 2 verbal anchors at the extrema (Fig. 18).

The third judgement required in Part A was a source typology of sounds ‘noticed’ during each stimulus. The intention of this aspect of the experiment was to obtain noticeability of UAS sounds indirectly, while avoiding directing attention towards the object of interest—this approach contrasts with classical signal detection tasks such as n-interval forced choice, which typically directly ask participants to indicate when they have detected the specific source of interest to the study. In this case, since the experiment information avoided explicitly mentioning UAS (see ‘Participants’ section), and the acoustic environment sound recording sections did not include audible aircraft movements (see Stimuli section), a judgement was classified as ‘UAS noticed’ in one of two ways (refer to Fig. 18): (i) if participants indicated having noticed an aircraft (by checking the corresponding source box); or (ii), if participants checked the ‘other’ source box and the associated description entered suggested that it was likely the UAS source had been detected (when present). In the latter case, descriptions that were assigned a ‘UAS noticed’ classification included examples such as “light/faint aircraft or machinery noise”, “drone or chainsaw”, “lawnmower”, “bees”, “buzzing insect”, and “garden equipment”.

For Part B, only the affective responses (SAM and annoyance) were included in the corresponding GUI, as the combination of the (quieter) CUPenv with the two highest UAS sound level intervals meant the UAS sounds were always clearly noticeable, when present in the scene.

The analysis presented herein (see ‘Results’ section) has been focussed on the annoyance and noticeability judgements; analysis of the other affective responses collected may be presented in future research.

### Pre-analysis data checking

The test data were initially examined for potential anomalies in the participant judgements that might indicate (for example) miscomprehension of the instructions. For the affective responses, the proportion of response outliers was calculated for each participant based on applying Tukey’s interquartile range (‘H-spread step’) criterion^50^ to the data separately for each experiment stimulus. From this analysis, the highest proportion of outliers within the total number of responses per participant was 12%, but the large majority (33 participants) had outlier proportions of 2% or less. On this basis, none of the participant affective response data were considered sufficiently anomalous to consider further action.

For the noticeability data, it was noted that some of the ‘ambient environment only’ stimuli analysed according to the approach set out in the Participants Judgements subsection had ‘UAS noticed’ classifications, which indicated that some sound within the scene may have been interpreted as aircraft-like or another sound similarly classified as indicating ‘UAS noticed’ had been heard. During further analysis (described below), it was also identified that aggregated noticeability (i.e., proportional ‘UAS noticed’ across participants) correlated well with partial loudness of the UAS components of the stimuli (see Results section). This observation was used in combination with aural reviews of the binaural stimuli recordings to identify occurrences when UAS were classified as ‘UAS noticed’ but would not be expected to have been noticeable, and, conversely, when the stimulus was classified as ‘UAS not noticed’, but would be expected to have been clearly noticeable. Occurrences of the former all corresponded either with the ‘ambient environment only’ stimuli, or with BCSenv scenes featuring the lowest UAS sound level intervals, and only when the partial loudness for the UAS component was close to 0 sones. Unexpected occurrences of ‘UAS not noticed’ corresponded with only the highest UAS sound levels within the BCSenv or the top two UAS sound levels within the CUPenv. This analysis identified 11 noticeability participant datasets with proportions of anomalous judgements that exceeded 40% for at least one of the noticed/not noticed criteria, ranging up to 100%—these cases also had a ‘UAS noticed’ classification for the ‘ambient environment only’ BCSenv scene. The noticeability datasets for these 11 participants were therefore considered unreliable and omitted from further analysis of this outcome variable, leaving 30 participant datasets remaining for noticeability analysis.

### Statistical analysis

Formal statistical analyses have been undertaken using the following software tools:

- Non-parametric effects estimation analysis: Python package DABEST-python (2024.03.29)
- Within-subjects ANOVA: JASP (0.19.0)
- Correlation analysis: Python packages pandas (2.1.4) and Pingouin (0.5.4)
- Power analysis: G*Power (3.1.9.7)

In view of cautionary advice concerning the application of Fisher-Neyman-Pearson ‘null hypothesis significance testing’-based approaches to frequentist statistical analysis^51–57^, the approach taken here focuses on ‘estimation statistics’ techniques^58–61^.

### Sound quality analysis

For the sound quality analyses, the following models and parameters were employed [as implemented in ArtemiS (16.0) software, unless otherwise noted]:

- Loudness: calculated according to the Sottek Hearing Model for binaural loudness as defined in ECMA-418-2:2022^62^ (with the time-aggregated values obtained using a ‘power average’ approach suggested from perceptual results^63^).
- Sharpness: calculated according to the model by Aures^64^ (see Supplementary Note 2 in the Supplementary information for further details) using the Moore-Glasberg-Schlittenlacher model for loudness as defined in ISO 532-3^65^ as input, with time-aggregation applied as the 95^th^ percentile (the ‘5% exceeded’ value), and the maximum value taken from the left/right channels (as suggested in ISO/TS 12913-3:2019^40^—for the experiment data in this case, the left/right values in the binaural recordings were very similar for all metrics).
- Tonality: derived from calculations using the Sottek Hearing Model for time-dependent specific tonality as defined in ECMA-418-2:2022^62^, with integration over critical bands applied to obtain time-dependent overall tonality, instead of the standardised approach of taking the spectral maximum tonality at each time step (see Supplementary Note 3 in the Supplementary information for further explanation). This was followed by time aggregation applied as the 95^th^ percentile, and the maximum value then taken from the left/right channels^40^.
- Roughness: calculated according to the model described by Fastl^66^ and Fastl and Zwicker^67^ (as implemented by MathWorks^68^, using ISO 523-1^69^ loudness as input), with time-aggregation applied as the 95^th^ percentile, and the maximum value taken from the left/right channels^40^.
- Fluctuation strength: calculated from the model of Osses Vecchi et al.^70^ (as implemented in the SQAT toolbox^71^—see Supplementary Note 4 in the Supplementary information for further details), with time aggregation applied as the 90^th^ percentile (the ‘10% exceeded’ value), and the maximum value taken from the left/right channels^40^.
- Impulsiveness: calculated according to the Sottek Hearing Model for time-dependent impulsiveness^72,73^, with time-aggregation applied as the arithmetic mean, and the maximum value taken from the left/right channels^40^.

The binaural HATS recordings were equalised prior to processing sound quality metrics, to allow for outer ear filtering stages in the psychoacoustic models.

## Supplementary information

Supplementary Information

## Data Availability

The datasets generated and analysed during the current study will be made publicly available via the project website (https://www.refmap.eu) in accordance with the project open access commitments and data management plan. Data will also be made available by the corresponding author on reasonable request, subject to project governance approval.
