# Supplementary material for: Human perception and response to sound from unmanned aircraft systems within ambient acoustic environments
Source: NPJ Acoust. 2025 Feb 12;1(1):2. doi: 10.1038/s44384-024-00001-6 (PMC11841372; doi:10.1038/s44384-024-00001-6)
Supplement: Supplementary file 1 — Supplementary Information [file 44384_2024_1_MOESM1_ESM.pdf]

Human perception and response to sound from unmanned aircraft systems within ambient acoustic environments

Michael J B Lotinga[1*], Marc C Green[1] and Antonio J Torija[1]

[1*]Acoustics Research Centre, University of Salford, 43 Crescent, Salford, M5 4WT, Greater Manchester, UK.

*Corresponding author(s). E-mail(s): m.j.lotinga@edu.salford.ac.uk;
Contributing authors: m.c.green@salford.ac.uk; a.j.torijamartinez@salford.ac.uk;

Abstract

Potential opportunities for unmanned aircraft systems (UAS) to offer societal benefits are accompanied by noise impact risks. Accordingly, it is important to develop greater understanding of perception and response to UAS sound. A laboratory listening experiment was undertaken to address this aim by investigating psychoacoustics of UAS sound exposure. The experiment incorporated contextual auditory and soundscape factors by embedding spatially-rendered UAS sounds within urban acoustic environments. The UAS covered varying aircraft designs, operating modes and numbers of flights. The experiment was focussed on determining noticeability and noise annoyance. The results indicate that annoyance responses were influenced by UAS type, operational mode, sound characteristics, quantities of flights, and the ambient acoustic environments in which UAS events occurred. Annoyance also appeared to have associations with personal attitude towards advanced air mobility technology, and with classification of residence area. Noticeability appeared to be influenced by UAS type, operating mode, loudness and ambient environment.

Supplementary information

Supplementary Note 1

The confirmatory aspects of our analysis are arguably closer in execution to what Fife and Rodgers [1] (after Tukey [2]) describe as ‘rough’ confirmatory data analysis (CDA), i.e., methods of evaluating hypotheses with an objective of discovery and theory development. This is distinct from their proposals for ‘strict’ CDA, the objective of which is to confirm/refute specific patterns established from previous results using a rigid, predefined testing framework. The analysis presented here also includes the use of analysis of variance (ANOVA), which is a tool more associated with ‘strict’ CDA, but complements the graphical approach with a more traditional perspective on the data. See Fife and Rodgers [1] and Fife [3] for detailed discussions, and useful recommendations for methodological approaches aiming to address the so-called ‘replication crisis’ in scientific research.

Supplementary Note 2

This sharpness model is based on a modification of the model developed by von Bismarck [4]. The modification accounts for varying loudness between stimuli, and was also tested using a wider range of sound characters than had been employed by von Bismarck [4] in developing the sharpness model kernel. The Aures [5] model is therefore considered to be more suitable for use with sounds spanning a range of loudnesses [6].

Supplementary Note 3

The ECMA-418-2:2022 [7] standardised method emphasises the highest tonality identified in the spectrum, and as such does not currently appear to adequately account for sounds that comprise multiple complex tonal harmonics. One alternative approach suggested in such situations [8] is to assess 'tonal loudness', which is also defined in ECMA-418-2:2022 [7]. However, tonal loudness (in sones) can naturally be expected to be closely correlated with total loudness. Another possible approach (suggested and taken here) is to integrate tonality over the critical bands, but with recalibration of the integrated result such that the reference value of 1 tu for a 1 kHz sinusoid at 40 dB is maintained. Our approach has the dual benefits of reducing the correlation with total loudness and retaining separate units for tonality.

Supplementary Note 4

This model for fluctuation strength is based on a model for roughness described by Daniel and Weber [9], which itself is an enhanced implementation of the roughness model developed by Aures [10].

Supplementary tables

Supplementary Table 1 Part A results ($n = 41$): Annoyance effects by UAS L_{Aeq} and ambient environment (within-subjects paired differences standardised effect sizes with 95% confidence intervals from bootstrap sampling over 5000 iterations)

Amb. env.	UAS L_{Aeq}		Sample size (responses)	Cohen's d		Permutation p-value[2]
	Control	Test		Value	95% conf. int.[1]	
Park	42	48	369	0.455	[0.344, 0.569]	<0.001
	48	54	369	0.375	[0.267, 0.482]	<0.001
	54	60	369	0.401	[0.301, 0.503]	<0.001
Street	42	48	369	0.155	[0.066, 0.244]	<0.001
	48	54	369	0.162	[0.068, 0.256]	<0.001
	54	60	369	0.404	[0.307, 0.512]	<0.001

UAS: unmanned aircraft system

[1]Bias-corrected and accelerated.

[2]The calculated values are adjusted to compensate for the potential bias caused by permutations resulting in p-values of 0 using an integral approximation approach proposed by Phipson and Smyth [11].

Supplementary Table 2 Part A results ($n = 41$): Annoyance effects by UAS type and ambient environment (within-subjects paired differences standardised effect sizes with 95% confidence intervals from bootstrap sampling over 5000 iterations)

Amb. env.	UAS L_{Aeq}	UAS type		Sample size (responses)	Cohen's d		Permutation p-value[2]
		Control	Test		Value	95% conf. int.[1]	
Park	All	H520	M300	492	−0.143	[−0.225, −0.060]	0.006
	All	H520	T150	492	−0.178	[−0.261, −0.094]	0.001
Street	All	H520	M300	492	−0.035	[−0.108, −0.041]	0.351
	All	H520	T150	492	−0.080	[−0.015, 0.008]	0.030
Park	60 dB	H520	M300	123	−0.385	[−0.570, −0.198]	<0.001
	60 dB	H520	T150	123	−0.306	[−0.498, −0.124]	0.002
Street	60 dB	H520	M300	123	−0.093	[−0.275, −0.075]	0.315
	60 dB	H520	T150	123	−0.142	[−0.281, 0.016]	0.039

UAS: unmanned aircraft system

[1]Bias-corrected and accelerated.

[2]The calculated values are adjusted to compensate for the potential bias caused by permutations resulting in p-values of 0 using an integral approximation approach proposed by Phipson and Smyth [11].

Supplementary Table 3 Part A results ($n = 41$): Annoyance effects by UAS operation and ambient environment (within-subjects paired differences standardised effect sizes with 95% confidence intervals from bootstrap sampling over 5000 iterations)

Amb. env.	UAS L_{Aeq}	UAS operation		Sample size (responses)	Cohen's d		Permutation p-value[2]
		Control	Test		Value	95% conf. int.[1]	
Both	All	Flyby	Landing	984	0.125	[0.070, 0.181]	<0.001
	All	Flyby	Takeoff	984	0.177	[0.119, 0.236]	<0.001
Both	60 dB	Flyby	Landing	246	0.139	[0.012, 0.264]	0.038
	60 dB	Flyby	Takeoff	246	0.329	[0.204, 0.463]	<0.001
Park	All	Flyby	Landing	492	0.143	[0.065, 0.228]	<0.001
	All	Flyby	Takeoff	492	0.175	[0.092, 0.261]	<0.001
Street	All	Flyby	Landing	492	0.111	[0.032, 0.189]	0.005
	All	Flyby	Takeoff	492	0.185	[0.103, 0.271]	<0.001

UAS: unmanned aircraft system

[1]Bias-corrected and accelerated.

[2]The calculated values are adjusted to compensate for the potential bias caused by permutations resulting in p-values of 0 using an integral approximation approach proposed by Phipson and Smyth [11].

Supplementary Table 4 Part A results ($n = 41$): Annoyance effects for within-subjects ANOVA (between-subjects factors and covariates model) — between-subjects effects summary

	F-statistic	Degrees of freedom		p-value	Effect size
Factors		factor	residuals		η^2_{p}
AOR soundscape character	1.502	3	26	0.237	0.148
AAM attitude	1.467	3	26	0.247	0.145
Age	2.276	1	26	0.143	0.080
AOR classification	0.751	2	26	0.482	0.055
Noise sensitivity	0.517	1	26	0.478	0.020
PANAS negative	0.259	1	26	0.615	0.010
PANAS positive	0.155	1	26	0.697	0.006
Sex	0.002	1	26	0.968	$<.001$

AAM: advanced air mobility, AOR: area of residence, PANAS: positive and negative affect schedule

Supplementary Table 5 Part B results ($n = 42$): Annoyance effects by UAS event quantity and type (within-subjects paired differences standardised effect sizes with 95% confidence intervals from bootstrap sampling over 5000 iterations)

UAS L_{Aeq}	UAS type	UAS event quantity		Sample size (responses)	Cohen's d		Permutation p-value[2]
		Control	Test		Value	95% conf. int.[1]	
54 dB	Both	1	3	84	0.384	[0.199, 0.582]	<0.001
	Both	3	5	84	0.159	[−0.036, 0.329]	0.091
	Both	5	9	84	−0.067	[−0.216, 0.067]	0.393
60 dB	Both	1	3	84	0.530	[0.283, 0.772]	<0.001
	Both	3	5	84	0.122	[−0.084, 0.316]	0.198
	Both	5	9	84	0.198	[0.050, 0.356]	0.011
54 dB	H520	1	3	42	0.275	[0.032, 0.517]	0.024
	H520	3	5	42	0.239	[0.030, 0.458]	0.028
	H520	5	9	42	0.000	[−0.193, 0.175]	0.912
54 dB	T150	1	3	42	0.502	[0.218, 0.806]	0.003
	T150	3	5	42	0.077	[−0.247, 0.337]	0.569
	T150	5	9	42	−0.131	[−0.355, 0.065]	0.202
60 dB	H520	1	3	42	0.458	[0.119, 0.794]	0.009
	H520	3	5	42	0.148	[−0.149, 420]	0.284
	H520	5	9	42	0.272	[0.075, 0.510]	0.005
60 dB	T150	1	3	42	0.604	[0.219, 0.963]	0.001
	T150	3	5	42	0.093	[−0.180, 0.353]	0.540
	T150	5	9	42	0.134	[−0.082, 0.366]	0.187

UAS: unmanned aircraft system

[1]Bias-corrected and accelerated.

[2]The calculated values are adjusted to compensate for the potential bias caused by permutations resulting in p-values of 0 using an integral approximation approach proposed by Phipson and Smyth [11].

Supplementary Table 6 Part B results ($n = 42$): Annoyance effects for within-subjects ANOVA (between-subjects factors and covariates model) — between-subjects effects summary

Factors	F-statistic	Degrees of freedom factor	Degrees of freedom residuals	p-value	Effect size η^2_p
AOR classification	3.687	2	27	0.038	0.215
AAM attitude	1.237	3	27	0.316	0.121
AOR soundscape character	1.062	3	27	0.382	0.106
PANAS negative	2.900	1	27	0.100	0.097
Age	1.091	1	27	0.306	0.039
Sex	0.338	1	27	0.566	0.012
PANAS positive	0.220	1	27	0.642	0.008
Noise sensitivity	0.099	1	27	0.755	0.004

AAM: advanced air mobility, AOR: area of residence, PANAS: positive and negative affect schedule

Supplementary Table 7 Parts A & B results ($n = 42$): Annoyance effects by between-subjects factors (between-subjects differences standardised effect sizes with 95% confidence intervals from bootstrap sampling over 5000 iterations)

Factor	Categories Control	Categories Test	Sample size (responses) Control	Sample size (responses) Test	Cohen's d Value	Cohen's d 95% conf. int.[1]	Permutation p-value[2]
AAM attitude	Supportive	Ambivalent	1034	768	0.321	[0.230 0.416]	<0.001
	Supportive	Concerned	1034	658	0.262	[0.164, 0.359]	<0.001
	Supportive	Neutral	1034	1410	0.526	[0.442, 0.608]	<0.001
Area of residence	Urban	Sururban	2538	1238	0.322	[0.258, 0.384]	<0.001
	Urban	Rural	2538	94	−0.008	[−0.212, 0.203]	0.934

AAM: advanced air mobility

[1] Bias-corrected and accelerated.

[2] The calculated values are adjusted to compensate for the potential bias caused by permutations resulting in p-values of 0 using an integral approximation approach proposed by Phipson and Smyth [11].

Supplementary figures

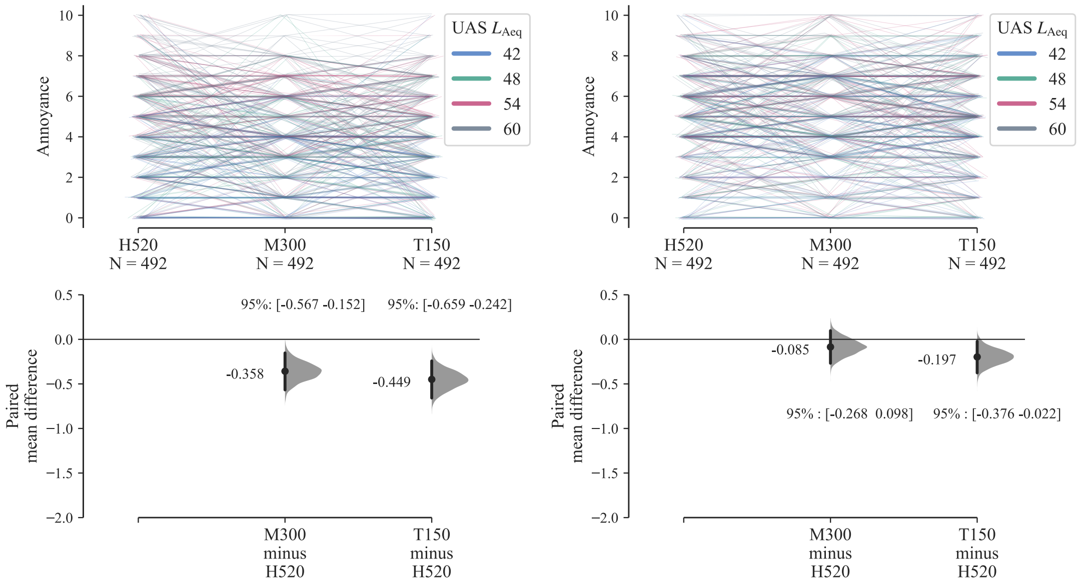

Supplementary Figure 1 Part A results ($n = 41$): Annoyance effects by UAS type and ambient environment; left, calm urban park; right, busy city street (Cumming estimation plots for within-subjects paired differences, effect size as paired mean difference with 95% confidence intervals from bootstrap sampling over 5000 iterations, line plot data points xy-jittered)

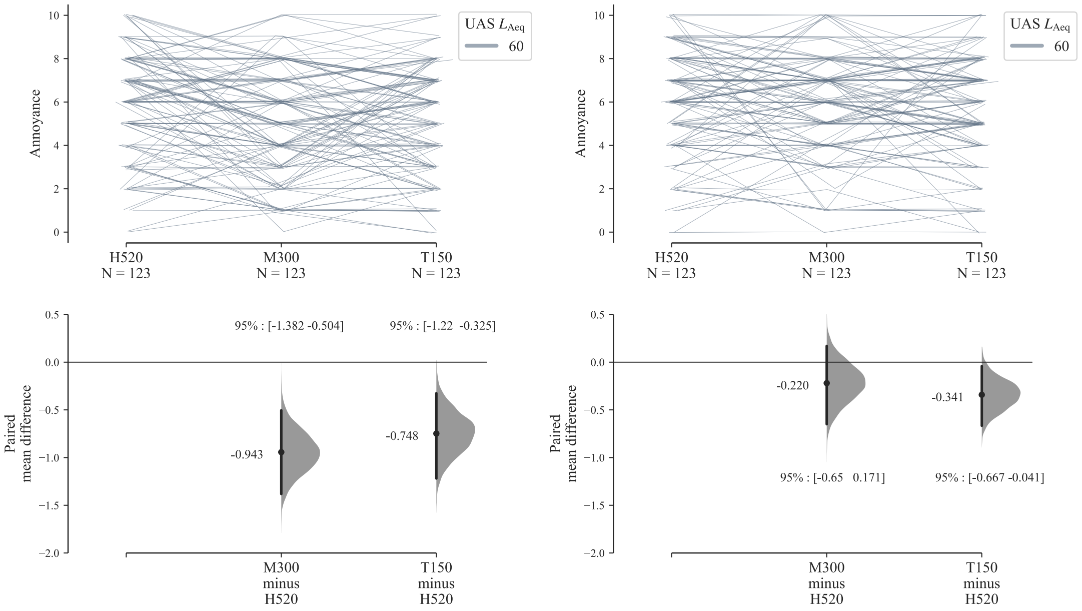

Supplementary Figure 2 Part A results ($n = 41$): Annoyance effects by UAS type and ambient environment, filtered for stimuli with UAS L_{Aeq} 60 dB; left, calm urban park; right, busy city street (Cumming estimation plots for within-subjects paired differences, effect size as paired mean difference with 95% confidence intervals from bootstrap sampling over 5000 iterations

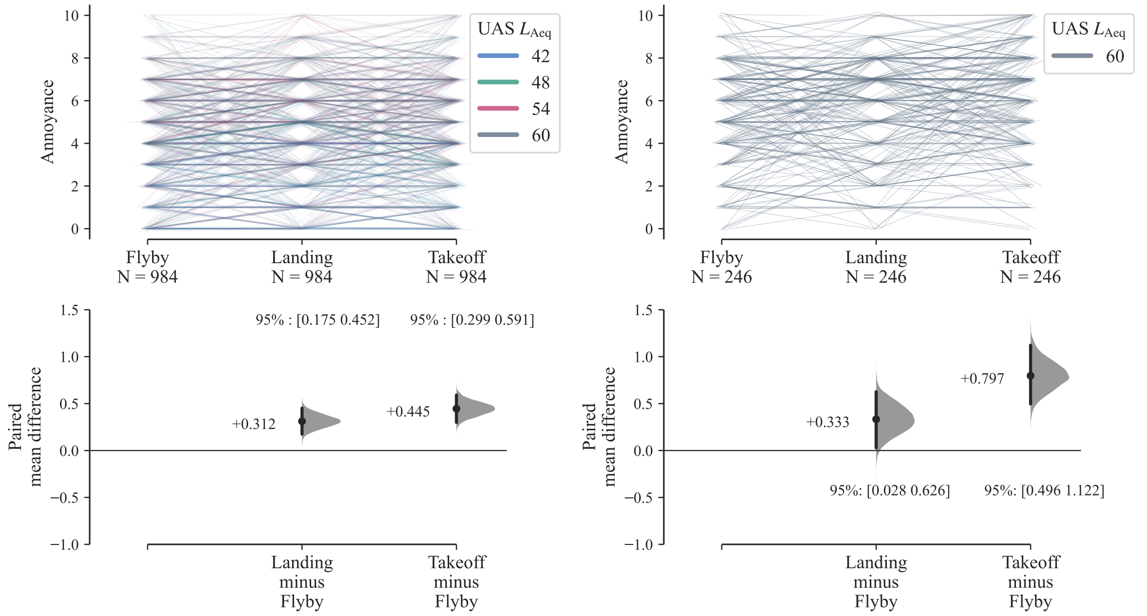

Supplementary Figure 3 Part A results ($n = 41$): Annoyance effects by UAS operation; left, all UAS L_{Aeq} intervals; right, UAS L_{Aeq} 60 dB (Cumming estimation plots for within-subjects paired differences, effect size as paired mean difference with 95% confidence intervals from bootstrap sampling over 5000 iterations, line plot data points xy-jittered)

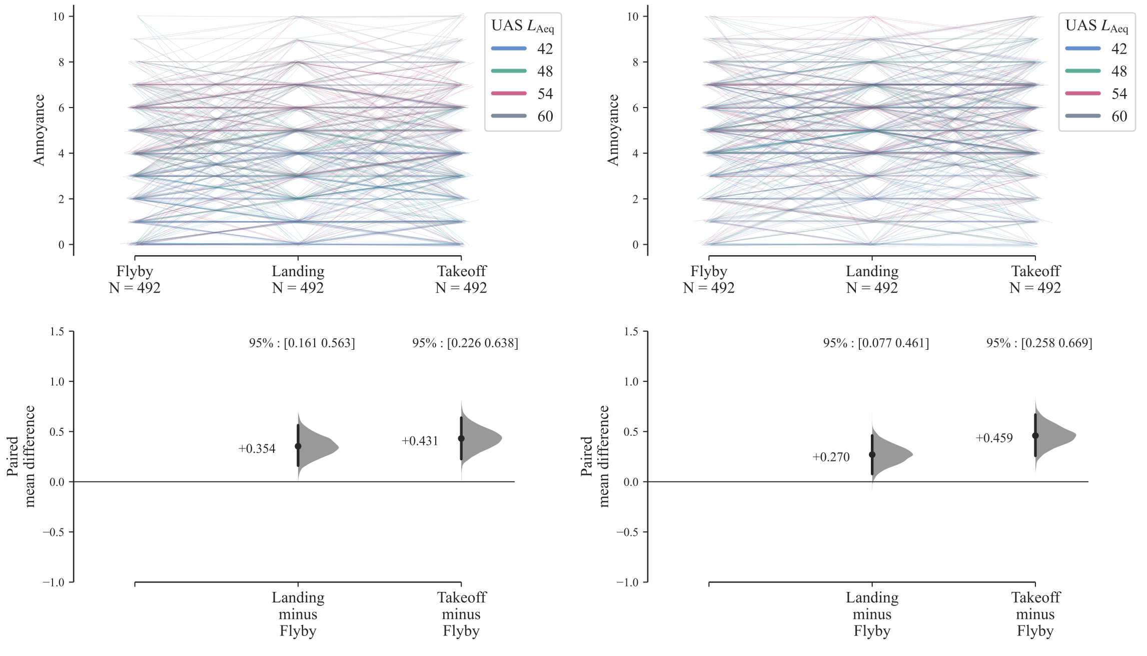

Supplementary Figure 4 Part A results ($n = 41$): Annoyance effects by UAS operation and ambient environment; left, calm urban park; right, busy city street (Cumming estimation plots for within-subjects paired differences, effect size as paired mean difference with 95% confidence intervals from bootstrap sampling over 5000 iterations

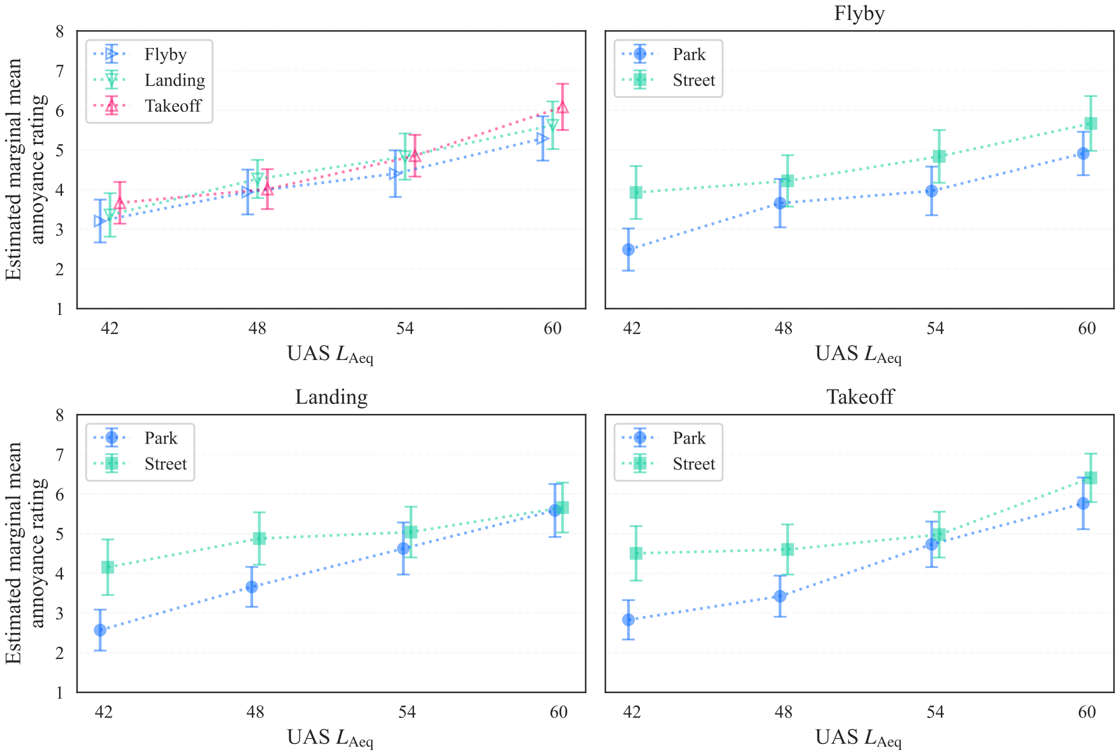

Supplementary Figure 5 Part A results ($n = 41$): ANOVA interaction analysis — ambient environment × UAS L_{Aeq} × operation (estimated marginal means with 95% confidence intervals)

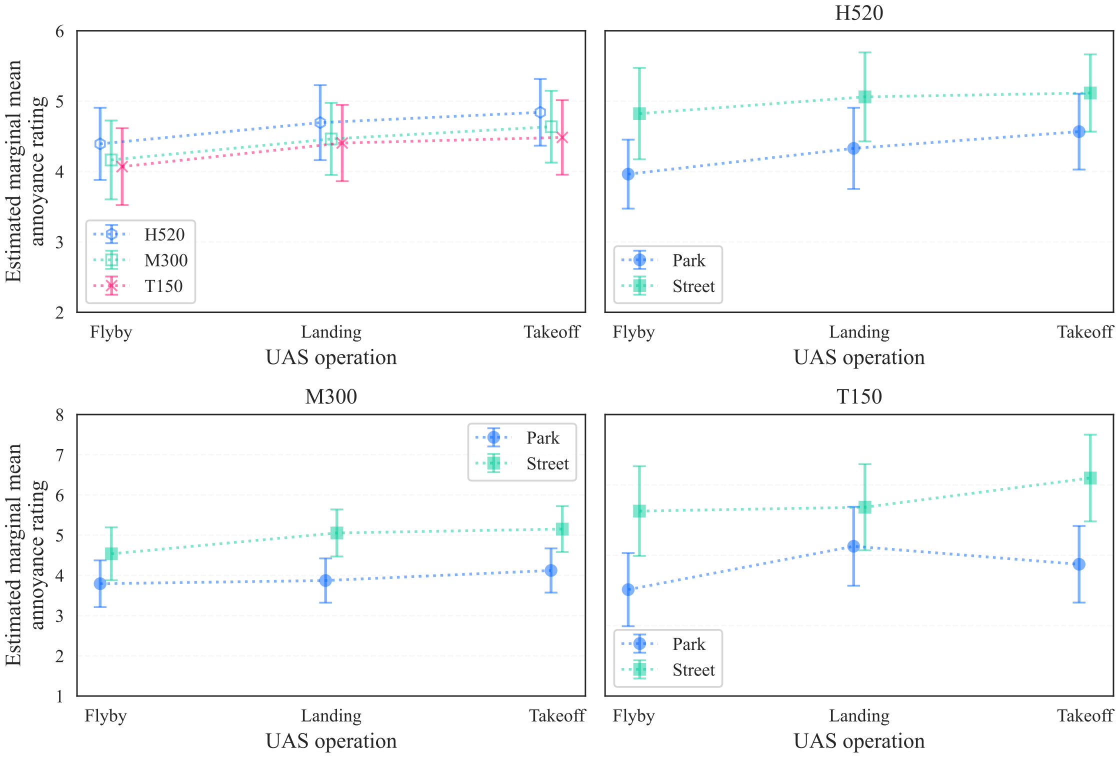

Supplementary Figure 6 Part A results ($n = 41$): ANOVA interaction analysis — ambient environment × UAS type × operation (estimated marginal means with 95% confidence intervals)

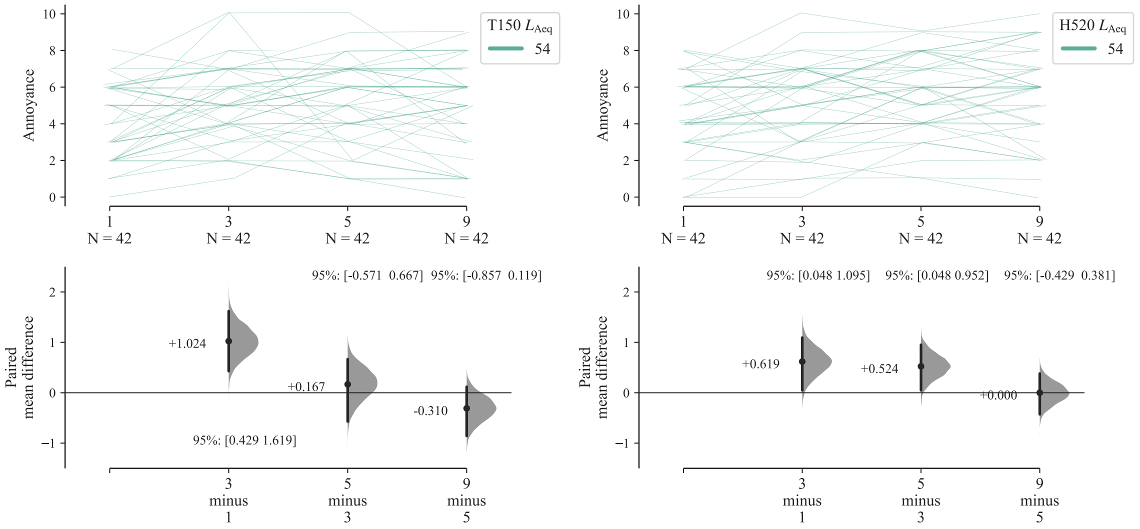

Supplementary Figure 7 Part B results ($n = 42$): Annoyance effects by UAS event quantity and type with UAS at 54 dB L_{Aeq}; left, T150; right, H520 (Cumming estimation plots for within-subjects paired differences, effect size as sequential paired mean difference with 95% confidence intervals from bootstrap sampling over 5000 iterations, line plot data points xy-jittered)

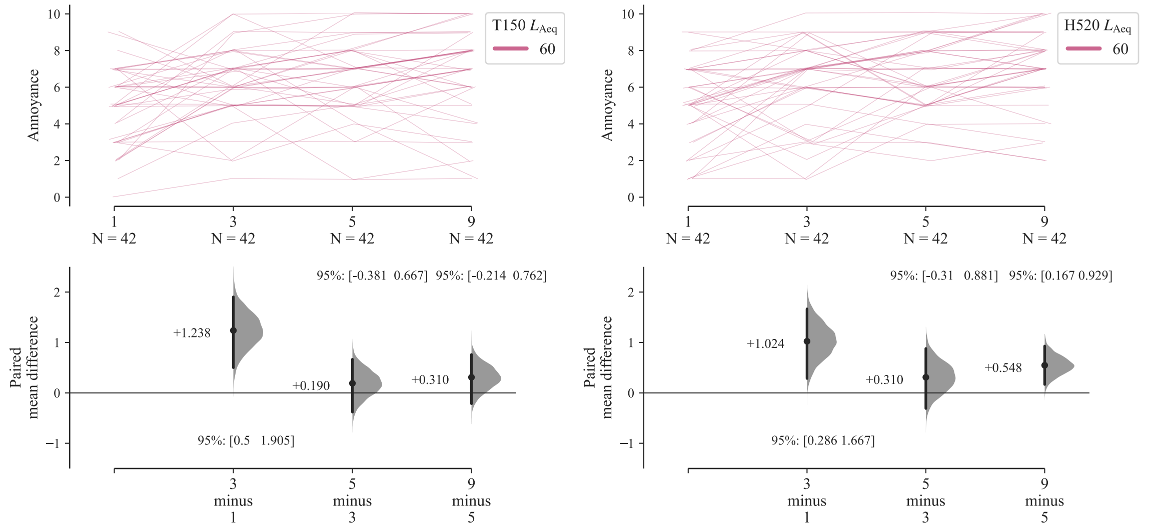

Supplementary Figure 8 Part B results ($n = 42$): As Supplementary Figure 7, for stimuli with UAS at 60 dB L_{Aeq}

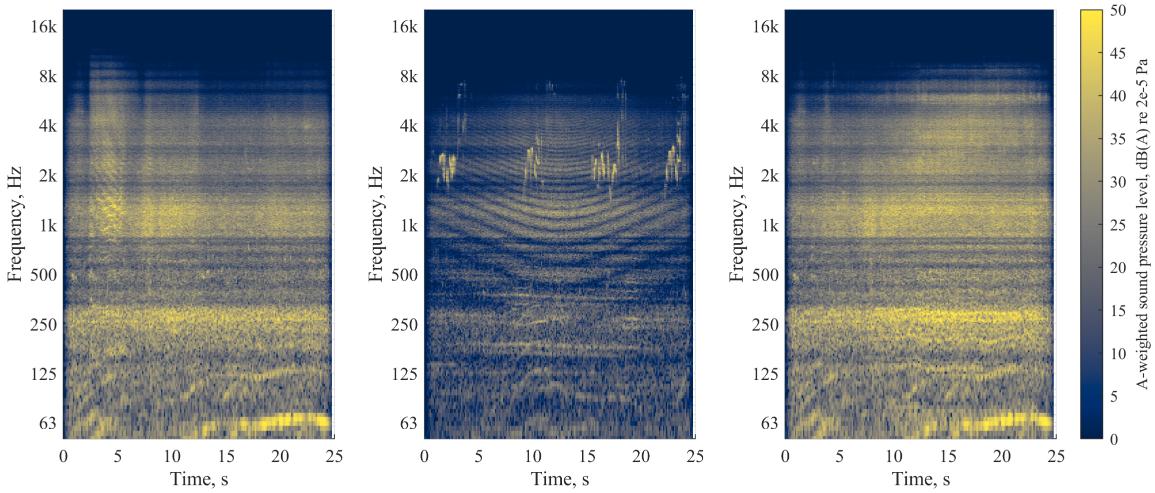

Supplementary Figure 9 Example spectrograms of Part A stimuli (binaural recordings at the participant position, incoherently summed); left: busy city street with H520 takeoff; middle: calm urban park with M300 flyby; right: busy city street with T150 landing (Δf 4 Hz, Δt 1/8 s, Hann-windowed 50% overlap)

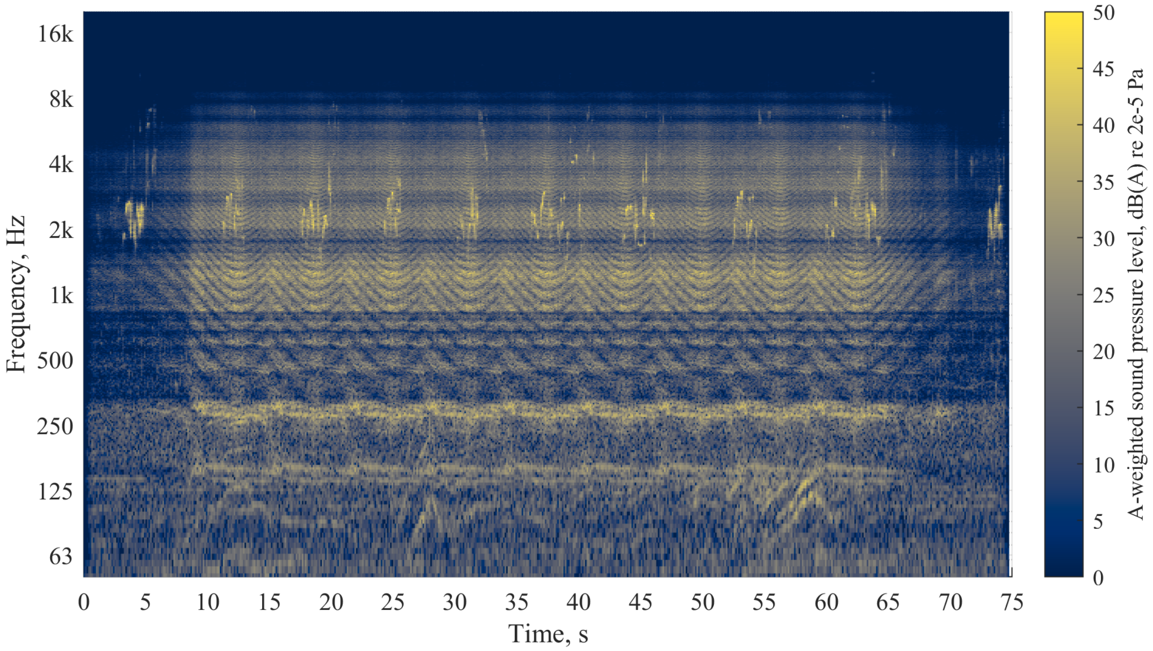

Supplementary Figure 10 Example spectrogram of Part B stimulus (binaural recording at the participant position, incoherently summed); calm urban park with 9× M300 flyby events (Δf 4 Hz, Δt 1/8 s, Hann-windowed 50% overlap)

References

[1] Fife, D.A., Rodgers, J.L.: Understanding the exploratory/confirmatory data analysis continuum: Moving beyond the “replication crisis”. American Psychologist **77**(3), 453–466 (2022) https://doi.org/10.1037/amp0000886

[2] Tukey, J.W.: Exploratory data analysis as part of a larger whole. In: Office, U.A.R. (ed.) Proceedings of the Conference on the Design of Experiments in Army Research Development and Testing (18th) Held at Aberdeen Proving Ground, Maryland, on 25 – 27 October 1972. (Part 1). National Technical Information Service, US Department of Commerce, Springfield, US (1973). https://apps.dtic.mil/sti/citations/tr/AD0776910

[3] Fife, D.: The eight steps of data analysis: A graphical framework to promote sound statistical analysis. Perspectives on Psychological Science **15**(4), 1054–1075 (2020) https://doi.org/10.1177/1745691620917333

[4] von Bismarck, G.: Sharpness as an attribute of the timbre of steady sounds. Acustica **30**(3), 159–172 (1974)

[5] Aures, W.: Berechnungsverfahren für den sensorischen Wohlklang beliebiger Schallsignale (Calculation method for the sensory euphony of arbitrary sound signals). Acta Acustica united with Acustica **59**(2), 130–141 (1985)

[6] HEAD Acoustics: Application Note: Psychoacoustics I — Loudness and sharpness calculation. Technical Report 02/18 (2018). https://web.archive.org/web/20240625202506/https://cdn.head-acoustics.com/fileadmin/data/global/Application-Notes/SVP/Psychoacoustic-Analyses-I_e.pdf

[7] Ecma International: ECMA-418-2:2022 Psychoacoustic metrics for ITT equipment — Part 2 (models based on human perception). Technical report (2022). https://www.ecma-international.org/publications-and-standards/standards/ecma-418/

[8] Sottek, R.: Psychoacoustic standards using the Sottek Hearing Model and their applications. University of Salford (2024)

[9] Daniel, P., Weber, R.: Psychoacoustical roughness: Implementation of an optimized model. Acta Acustica united with Acustica **83**(1), 113–123 (1997)

[10] Aures, W.: Ein Berechnungsverfahren der Rauhigkeit (A calculation method for roughness). Acta Acustica united with Acustica **58**(1), 268–281 (1985)

[11] Phipson, B., Smyth, G.K.: Permutation p-values should never be zero: Calculating exact p-values when permutations are randomly drawn. Statistical Applications in Genetics and Molecular Biology **9**(1) (2010) https://doi.org/10.2202/1544-6115.1585
